# Modified Fruit Peels as Sustainable Sorbents for Removing Heavy Metal Ions in Wastewater

**DOI:** 10.1155/tswj/4088630

**Published:** 2026-10-23

**Authors:** Abiegael N. Kiiru, George S. Nyamato, Joanne Ogunah, Stephen O. Ojwach

**Affiliations:** ^1^ Department of Physical Sciences University of Embu Embu Kenya embuni.ac.ke; ^2^ School of Chemistry and Physics University of KwaZulu-Natal Pietermaritzburg South Africa ukzn.ac.za

**Keywords:** biosorption, fruit peel waste, green technology, heavy metal ions, surface modification

## Abstract

There has been a continuous surge in the production of fruit peel waste worldwide, driven by improved dietary habits and rapid population growth. The management of these wastes through conventional approaches, such as landfilling and incineration, presents serious environmental challenges. This is because of reduced land availability, the release of toxic gases during open‐air burning, and the slow decomposition of these wastes, which releases methane, causing climatic changes. Reports on the presence of heavy metal ions in wastewater discharged from industries have been conducted, indicating their potential threats to both human health and the environment. High costs often limit conventional approaches to removing heavy metal ions; thus, fruit peel waste‐based biosorbents offer a low‐cost alternative. Utilizing fruit peel waste as a biosorbent for the removal of heavy metal ions promotes a circular economy approach to achieving the Sustainable Development Goals. Therefore, this review examines the composition of fruit peel waste and its potential application as a biosorbent for removing heavy metal ions. Additionally, the findings contribute to an understanding of the mechanisms involved in the biosorption process, the modification strategies employed to enhance biosorption capacity, the regeneration ability of biosorbents, and the potential application of the extract in the synthesis of various nanoparticles utilized in heavy metal ion removal.

## 1. Introduction

Water is an indispensable resource that sustains life on Earth and is vulnerable to pollution [1]. It plays a vital role in the world′s economic and social growth, as it is utilized in numerous industrial processes [2]. In this era of rapid industrialization, urbanization, and agricultural development, particularly in middle‐income and low‐income countries, the discharge of toxic industrial effluents, household, and agricultural wastes into water sources has reduced access to safe, clean drinking water [3]. According to the World Health Organization (WHO), 2.1 billion people globally lack access to safely managed drinking water, including 106 million who consume water from untreated sources [4]. In addition, freshwater resources are continuously strained due to their extensive use in various processes, prompting the search for new solutions to prevent their overexploitation [5]. An alternative water source for processes includes the effective treatment of wastewater generated from various activities [6]. The generation of wastewater from industrial activities, which contains a range of contaminants, has become a global challenge in its management and treatment [7]. Thus, effective methods for eliminating contaminants from wastewater are sought to provide clean, reusable water to attain the United Nations Sustainable Development Goal 6 (SDG6) [8].

Water pollutants are classified into organic, inorganic, and microbial contaminants, which render water unfit for human consumption and drastically impact the aquatic ecosystems [9, 10]. Unlike organic pollutants, many inorganic substances easily dissolve and linger longer in marine systems, degrading the pristine quality of water [2]. Typically, inorganic pollutants include heavy metals such as arsenic, mercury, lead, and cadmium, as well as mineral acids and complexes of inorganic substances that pose threats to aquatic life, human health, and the environment [11]. Among these inorganic pollutants, heavy metals are especially concerning due to their high toxicity, even at very low levels [12]. Their ionic state is the most toxic form, as it allows them to form complexes with other compounds that are difficult to biodegrade [13]. Moreover, heavy metal ions are highly soluble in water, allowing for easy absorption by aquatic organisms such as fish, resulting in bioaccumulation [14]. This eventually leads to biological magnification through the food chain as the toxicity concentration gradually increases [14].

Conventional methods, including chemical precipitation [15–18], ion exchange [19–21], membrane technology [22–25], and adsorption [26–28], are currently utilized to remove heavy metal ions from contaminated water. Adsorption is considered the most effective strategy for removing heavy metal ions as it offers several advantages over other techniques [29]. These advantages include its simple operation, lack of sludge‐related costs, selectivity, reusability potential, and its ability to effectively remove heavy metal ions at very low concentrations [30]. Moreover, adsorption offers many available adsorbent choices depending on the targeted heavy metal ions, costs, and the water quality requirements [31]. For example, commercial activated carbon has been utilized since the 20^th^ century for the elimination of heavy metal ions in wastewater treatment plants, mainly because of its porous structure, high surface area, chemical and thermal stability, and its high adsorption capacity [32]. However, its application is often limited by the high production and regeneration costs, low selectivity, and its regulated use in resource‐restrained environments, necessitating the need for low‐cost unconventional adsorbents [32]. The use of microbial biota, plant materials, or agricultural waste‐derived materials as unconventional adsorbents, entailed in biosorption, offers a lower‐cost alternative to adsorbents and more flexible sorption designs [33].

Recent research has shifted towards using low‐cost, nontoxic adsorbents derived from by‐products or waste materials, supporting the circular economy approach [34]. Agricultural wastes, particularly fruit peel waste, contain various bioactive compounds capable of adsorbing heavy metal ions [35]. These wastes are naturally composed of cellulose, hemicellulose, pectin, and lignin, which have functional groups with metal‐binding capacities [36]. These fruit peel wastes are mostly disposed of through landfilling, composting, and incineration, which pose serious environmental pollution issues [37]. Landfilling presents a societal challenge in municipal solid waste management, as fruit peel wastes biodegrade at a slower rate, accumulating extensively [36]. Their decomposition also releases methane, a greenhouse gas that contributes to global warming [38]. Moreover, burning of fruit peel wastes in open air releases toxic compounds harmful to both living organisms and the environment, while composting results in malodorous emissions [37]. Utilizing fruit peel wastes as biosorbents to remove heavy metal ions offers a sustainable solution for addressing both fruit peel waste disposal and water contamination, aligning with the circular economy principle of transforming waste into valuable materials.

A large number of recently published review articles (Table 1) have primarily focused on fruit peel wastes as biosorbents for various environmental pollutants. A summary of their use in their raw and modified forms has been discussed, but there remains a critical gap in their dual functionality. Apart from their direct application as biosorbents for heavy metal ions, their extracts can be employed as synthesis/modifying agents for various nanoparticles involved in green nanotechnology. This review addresses the knowledge gaps described in Table 1, by providing an integrated aspect in the valorization of fruit peel wastes for wastewater remediation and summarizes recent advances in fruit peel biosorbents. In this review, the composition of fruit peel wastes that makes them excellent biosorbents for the biosorption of heavy metal ions is presented, and the role of surface modification as a means to enhance their surface properties for the efficient elimination of heavy metal ions from wastewater is also elaborated. The main mechanisms involved in the biosorption process are also described, along with the kinetics and isotherm studies, and their potential for regeneration. Moreover, the use of fruit peel waste as a modifying agent for the biosynthesis of nanoparticles for the removal of heavy metal ions is also discussed.

**Table 1:** Recent review articles on fruit peel biosorbents.

Author	Review focus	Research gap relevant to the present review	Ref
Aguilar et al. (2022)	The review summarized studies published from 2010 to 2021 on the fruit peels used in Colombia as sustainable biosorbents for heavy metal ions.	Summarized only research studies published from 2010 to 2021.	[39]
Veronica and Ntumba (2024)	The review considered different physicochemical characteristics of vegetable and fruit peels that make them suitable raw materials for biosorbent production, their adsorption efficiencies, and regeneration potential.	Focused on both vegetable and fruit peel wastes.	[40]
		Specifically focused on the thermal transformation of the wastes.	
Fazila et al. (2024)	This review analyzed the potential utilization of agriculture‐based biosorbents for the removal of contaminants from toxic water/wastewater, as well as their mechanisms, adsorption efficiency, and regeneration ability.	Provided a concise mini‐review of the fruit peel adsorbents	[41]
		Incorporated the removal of other organic and inorganic pollutants	
Nyika and Dinka (2023)	This study reviewed reported works on the usability of different fruit biopeels as adsorbents for water pollutants. It discussed various physicochemical modifications, adsorption performance, and regeneration potential.	Focused on the both removal of both dyes and heavy metal ions	[42]
Oladipo et al. (2018)	Reviewed developments in the use of raw and modified adsorbents for the adsorption of antibiotics, pesticides, and endocrine disruptors from wastewaters	Particularly focused on organic pollutants without the inclusion of summarized studies on heavy metal ions	[43]
Present review	It discusses adsorption performance, fruit peel composition, modification strategies, adsorption mechanisms, kinetics, isotherms, regeneration, recent studies (2020–2026), and future research directions.	Provides an integrated perspective on the dual functionality of fruit peel wastes	
		Specifically focused on reported studies on heavy metal ions	
		Provides up‐to‐date research inputs	

## 2. Review Methodology

This study adopted a narrative review approach, integrating a systematic literature search with a critical narrative synthesis of the published works. The systematic search followed the PRISMA 2020 framework to ensure transparency and reproducibility in the identification, screening, eligibility assessment, and selection of relevant studies. Meta‐analysis was not performed because the selected studies exhibited methodological heterogeneity, including differences in fruit peel types, adsorbent preparation and modification techniques, target heavy metal ions, experimental conditions, and the adsorption models and performance indicators reported. Therefore, a structured narrative synthesis was used to compare adsorption performance, mechanisms, and research trends while identifying knowledge gaps and future research needs.

### 2.1. Search Strategy

The literature search was conducted using the four major science databases, including Scopus, Web of Science, Wiley Online Library, and Science Direct. The search included peer‐reviewed articles published from 2020 to 2026 to capture the most recent advances in the use of fruit peel wastes as sustainable sorbents for heavy metal ions in wastewater. This search strategy combined keywords related to fruit peel wastes, biosorption, and heavy metal ions using Boolean operators. The search terms were developed based on five concepts, which were fruit peel wastes, adsorption process, heavy metals, water treatment, and combined search. Keywords were combined within and across concepts in terms of Boolean operators (AND, OR) as shown in Table 2.

**Table 2:** Search keywords and Boolean operators.

Concept	Keywords	Boolean operators used across all databases
Fruit peel wastes	“fruit peel”, “fruit peel waste”, “fruit waste”, “fruit by‐products”, “fruit residues”, banana peel, orange peel, lemon peel, mango peel, pineapple peel, pomegranate peel, pomelo peel	(“fruit peel” OR “fruit peel waste” OR “fruit waste” OR “banana peel” OR “orange peel” OR “lemon peel” OR “mango peel” OR “pineapple peel” OR “pomegranate peel” OR “pomelo peel”)
Adsorption process	Adsorption, adsorbent, biosorption, biosorbents, sorption	(adsorption OR adsorbent OR biosorption OR biosorbent OR sorption)
Heavy metals	“heavy metal”, “heavy metal ions” lead, cadmium, chromium, copper, zinc, arsenic	(“heavy metal” OR “heavy metal ions” OR lead OR copper, OR cadmium OR arsenic OR zinc)
Water treatment	Wastewater, “wastewater treatment”, “synthetic solution”, effluent, contaminated water	(wastewater OR “wastewater treatment” OR “synthetic solution” OR effluent OR “contaminated water”)
Combined search	Fruit peel wastes + biosorption + Heavy metal ions + water treatment	(“fruit peel” OR “fruit waste” OR “banana peel” OR “orange peel”) AND (adsorption OR biosorption OR adsorbent) AND (“heavy metal ions” OR lead OR cadmium OR chromium OR copper) AND (wastewater OR “synthetic solution” OR effluent)

### 2.2. Eligibility Criteria

#### 2.2.1. Inclusion Criteria

The studies that were included had the following criteria:

• Peer‐reviewed journal published from 2020 to 2026.
• Journals published in English.
• Studies investigating fruit peel wastes as biosorbents.
• Studies conducted on the removal of heavy metal ions from aqueous solutions and wastewater.
• Experimental studies reporting biosorption performance, mechanisms, thermodynamics, kinetics, and isotherm models.
• Experimental studies reporting regeneration potential and industrial applicability of fruit peel biosorbents.
• Reviews that provided background information.
• Relevant studies from 2015–2020 that provided supporting information

#### 2.2.2. Exclusion Criteria

The literature studies excluded were nonpeer‐reviewed journals (such as conference reports and dissertations), informal collections, duplicate publications, studies lacking sufficient quantitative data, and studies not relevant to the use of fruit peel wastes were not included in this study.

### 2.3. Study Selection Process

The study selection process was guided by the four PRISMA stages: identification, screening, eligibility, and inclusion. A total of 1743 articles were identified, where 474 duplicates were removed, 1269 screened based on their titles and abstracts, 590 full‐texts were assessed against the inclusion/exclusion criteria and 236 studies were included in the study as illustrated in Figure 1.

**Figure 1:**
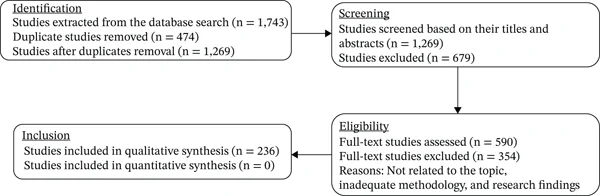
PRISMA flow diagram.

## 3. Heavy Metals

Heavy metals are described in literature as natural elements with a density above 5 g/cm^3^, an atomic mass greater than 20, and a density five times that of water [13]. They are classified as either essential or nonessential based on their toxicity. Essential heavy metals are necessary for human survival and agricultural productivity, whereas nonessential heavy metals have no biological role in humans and are not required for agriculture due to their acute and chronic toxic effects [44]. Essential heavy metals include copper, iron, nickel, and zinc, which are needed in trace amounts for various biochemical and physiological functions, but at excessive levels, can be highly toxic [45]. These elements act as cofactors for proteins and enzyme systems involved in DNA synthesis, cell differentiation, proliferation, and respiration [46]. Conversely, nonessential heavy metals, including lead, arsenic, cadmium, and mercury, have no physiological functions and are associated with deadly diseases such as cancer [45]. Excessive accumulation of both essential and nonessential heavy metals can be lethal; however, nonessential heavy metals are considered “the dark side of chemistry” because they cause toxicity even at low doses [47], making them the main focus for elimination from water sources.

Heavy metal ions are naturally occurring elements that enter water sources through natural processes or human activities, such as mining, untreated industrial discharges, reckless agricultural practices, and improper waste disposal [48]. Heavy metal ions, including Cd(II), Pb(II), Hg(II), and As(III) ions are among the most noxious, associated with poisoning to living organisms, even in lower doses [49]. The human exposure route to these metal ions mainly involves ingestion, inhalation, and dermal contact, which result in their accumulation in various body organs [46]. This exposure has been linked to chronic and acute toxicity that develops retardation, damaged kidneys due to neurotoxicity, various kinds of cancers, fragile bones, liver, and lung damage [50]. Additionally, exposure to heavy metal ions, such as lead in small amounts, is attributed to more than 1.5 million deaths globally due to cardiovascular effects [51]. This necessitates the need to efficiently eliminate these heavy metal ions from wastewater before their discharge and from drinking water before consumption by human beings.

### 3.1. Sources of Heavy Metal Ions in Water

The ongoing evolution of human activities, particularly with industrialization, has resulted in the generation of a significant volume of wastewater contaminated with these toxic elements [52]. The proliferation of heavy metal ions in water sources is associated with diverse processes, including industrial activities such as metal plating, mining, electroplating, petroleum refining, wood processing, and battery manufacturing [52]. Additionally, chemical processes like corrosion of pipe systems and metal equipment deterioration, release of agricultural wastes containing pesticides and chemical fertilizers, and improper waste disposal that results in leaching of the metals [53] have resulted in heavy metal‐contaminated water sources. Certain international organizations, including the WHO and the Environmental Protection Agency (EPA), have set permissible limits of heavy metal ions in water, and exposure beyond these limits is health‐threatening and is classified as carcinogenic [54]. Table 3 summarizes the toxic forms, prevalent exposure routes, sources, potential health effects, and the permissible limits of the most toxic heavy metal ions in water.

**Table 3:** The toxic form, prevalent exposure routes, potential health effects, and permissible limits of heavy metal ions.

Metal	Toxic form	Exposure route	Sources	Potential health effects	Permissible limits [ 55 ]	
					WHO	USEPA
Cadmium	Cd(II)	Inhalation	Zinc purification, batteries, alloys, pigments, plastic stabilizers, dyes, and paints containing cadmium [56]	Cancer, leukemia, and lung diseases; diseases related to the central nervous system, such as Alzheimer′s and Parkinson′s disease [56]	0.003	0.005
		Oral				
		Dermal				

Lead	Pb(II)	Inhalation	Battery industries, lead‐based paints, lead smelting, fertilizers and pesticides, metal plating, and urban waste [57]	Respiratory diseases, cardiovascular diseases, anemia, kidney failure, and hearing loss [57]	0.01	0.015
		Oral				

Arsenic	As(III)	Inhalation	Smelting of nonferrous metals, coal burning, chemical industries, cement production [58]	Cancer, epigenetic changes, kidney, skin, cardiovascular diseases, neurological disorders, and cognitive impairment [59]	0.01	0.01
		Oral				
		Dermal				

Chromium	Cr(VI)	Inhalation	Welding fumes, cement and steel production, coal and oil refining, metal and ore processing, leather tanning, dye and pigment production, agricultural fertilizers, and wood preservation [60]	Dermatitis, nasosinusal, kidney and liver problems, hematological and chromosomic aberrations [61]	0.05	0.1
		Dermal Contact				
		Oral				

Mercury	Hg(II)	Dermal	Cosmetic and medical products, coal‐fired power plants, coal combustion, incineration of waste, rechargeable batteries, electrical switches, paints, explosives, cement, and chemical production [62]	Cancer, endothelial dysfunction, gastric and vascular disorders, liver, kidney, and brain damage, hormonal imbalances, miscarriages and reproductive disorders, skin lesions, vision damage, and death [62]	0.006	0.002
		Oral				
		Inhalation of methyl‐mercury				

Human activities, especially industrial and improper agricultural practices, have increased the presence of toxic heavy metal ions in water sources [63]. Traditional methods used for removal can sometimes be ineffective at reducing these ions below permissible limits [8]. Therefore, research should focus on developing strategies that are not only efficient but also cost‐effective, eco‐friendly, and aligned with Sustainable Development Goals.

### 3.2. Heavy Metal Ions Removal From Water

Removing heavy metal ions from contaminated water is essential for the provision of safe and clean drinking water. The complex nature of wastewater, as well as the potential threat of heavy metal ions towards human and environmental health, has imposed the need for the use of various strategies to efficiently eliminate these inorganic pollutants. Treatment strategies are primarily classified under physical, chemical, or biological methods, as summarized in Table 4.

**Table 4:** Classification of heavy metal ions removal strategies.

Class	Technique	Practical applicability	Scalability	Cost	Environmental sustainability	Technological maturity	Ref
Physical	Adsorption	Most suitable for treating wastewaters with low heavy metal concentrations. Performance depends on adsorbent properties	Easily integrated into existing treatment systems	Based on the adsorbent type	High when adsorbents that can be reused are employed	Widely implemented in industrial wastewater treatment using conventional adsorbents	[64]
				Regeneration encourages cost‐effectiveness			
					Proper handling and disposal of spent adsorbent remains a concern		
	Membrane technology	Suitable for higher extraction efficiencies and metal ion recovery	Limited by membrane fouling and maintenance requirements	High due to initial capital investment, maintenance, and membrane replacement	Low chemical consumption encourages sustainability but membrane disposal still presses environmental concerns	Commercialized for industrial wastewater treatment	[22]
	Ion exchange	Ideal for selective metal ion removal and recovery of dissolved ions	Easily scalable but pretreatment is often required	High costs of resins regeneration costs are also high	Regeneration of chemicals promotes sustainability	Applied industrial applications, mostly for softening hard water	[65]

Chemical	Chemical precipitation	Utilized as a primary treatment process	Easily applied to large wastewater volumes	Depends on the type of the chemical precipitants	Generation of sludge limits sustainability	Extensively applied in wastewater treatment	[66]
	Coagulation and flocculation	Primarily used as a pretreatment step to enhance the treatment efficiency of subsequent steps	Suitable for continuous large‐scale operation.	Depends on chemical usage	The use of chemicals and sludge production limits sustainability	Employed in municipal and industrial treatment systems	[52]
	Solvent extraction	Applicable for recovering valuable metals	Process complexity limits scalability	High because of solvent use and operational requirements	Low due to solvent toxicity and handling concerns	Widely applied in hydrometallurgical operations	[67]

Biological	Bioremediation	Suitable wastewaters with low concentrations of ions	Scalability is influenced by environmental and operational conditions	Low because of minimal chemical and energy requirements	High owing to low environmental impact and minimal secondary waste generation	Limited industrial application	[68]
	Phytoremediation	Suitable for long‐term remediation of low‐contamination sites rather than industrial wastewater treatment.	Not easy to scale as it requires large land areas and extended treatment periods	Low operational and maintenance requirements	Sustainable due to minimal energy input and ecosystem restoration benefits.	Reported for environmental remediation but limited for industrial treatment systems.	[69]

The chemical transformation of heavy metal ions through chemical precipitation is widely applied in industries mainly because of its simple process control, wide temperature range applicability, and its low operation costs [66]. It involves precipitating heavy metal ions as hydroxides, carbonates, or sulfides through pH adjustment [70]. Hydroxide precipitation is the most utilized form of precipitation in industries, stemming from its cost‐effectiveness, simplicity, and the ability to adjust the pH [8]. However, it is often limited by its effectiveness in only large amounts of the precipitants, which increases costs and raises economic concerns [71]. Moreover, alternatives such as sulfide precipitation result in the emission of toxic hydrogen sulfide, which is a toxic environmental pollutant that contributes to air pollution [72]. Chemical precipitation also produces large amounts of hazardous sludge that cause secondary pollution, leading to higher treatment burdens [71].

Ion exchange is applied in wastewater treatment for the replacement of detrimental heavy metal ions with innocuous and ecologically benign ions from the solid ion exchanger [65]. It offers high selectivity, high removal efficiency, metal recovery, and water reuse [73]. Nevertheless, its application in industries is limited by fouling of the exchange matrix, which reduces its effectiveness, and it often results in lower removal efficiencies, particularly in highly concentrated solutions [74]. Membrane technology has been widely applied in the recent past for heavy metal ion removal from wastewater as a good substitute for traditional methods such as ion exchange and chemical precipitation, as chemical reagents are not required in the separation process [74]. It is also useful in water treatment because of its reduced energy consumption when compared with methods such as distillation, reduced solid waste production when compared to chemical‐based methods such as chemical precipitation, and its ease of integration with other processes [75]. Reverse osmosis is regarded as the most efficient membrane technique for water purification, mainly because of its high‐energy efficiency, simple construction, mechanical stability, and higher rejection levels [24, 76]. However, its application in industries is limited by its high operational costs [76]. Membrane fouling, caused by the accumulation of suspended solids, organic matter, microorganisms, or scaling compounds, remains a significant challenge in membrane technology, increasing costs and reducing overall efficiency due to reduced membrane lifespan [22]. Moreover, the struggle with low selectivity and demand for significant investment and operational expenses also presents additional challenges associated with membrane technology [74].

To address these challenges associated with the current techniques, adsorption remains a versatile and promising method for efficiently removing heavy metal ions from wastewater. Extensive research has explored the adsorptive removal of heavy metal ions using various adsorbents [29, 77–80]. Adsorption‐based wastewater treatment typically involves transferring heavy metal ions dissolved in water onto the surface of a porous solid adsorbent [77]. These interactions mainly depend on the nature of the adsorbent material, including the functional groups on its surface, and the type of heavy metal ions involved [77]. Over time, developing cost‐effective, selective, and easily regenerable adsorptive materials has made adsorption a highly effective strategy for reducing heavy metal ion concentrations to WHO permissible levels. This approach aligns well with the Sustainable Development Goals and promotes the circular economy.

## 4. Adsorption of Heavy Metal Ions

The adsorption process involves the transfer and retention of heavy metal ions from the bulk solution phase to the surface of a solid adsorbent [49]. It is one of the most effective traditional techniques applied in water and wastewater treatment plants because it is easy to implement and is cost‐effective. Additionally, its regeneration potential reduces the extra expenses associated with sludge removal [81]. It primarily depends on the adsorbate characteristics, including molecular weight, structure, size, charge, and solution concentration, as well as the adsorbent properties, such as size, surface area, surface charge, and surface functional groups, all of which are responsible for effective adsorption [49]. Apart from the adsorbate and adsorbent characteristics, parameters such as pH, contact time, adsorbent dosage, temperature, and the initial metal ion concentration greatly influence the adsorption process [82].

Depending on the nature of adsorbate‐adsorbent interactions, adsorption can occur through physical adsorption, arising from weak van der Waals forces, or through chemical adsorption, associated with chemical interactions between the ions and the adsorbent [83]. While physisorption is often characterized as reversible, weak, and endothermic, chemisorption is irreversible, more selective, and exothermic [84]. However, both mechanisms can occur simultaneously in practical water treatment applications, depending on the adsorbent′s properties and the nature of the solution [85].

Over the years, natural adsorbents such as clay and zeolites have proven effective in the removal of heavy metal ions from water sources. For instance, Azanfire et al. [26] reported high adsorptive performance of kaolinite‐based clay with removal efficiencies of over 98% for lead ions and 93% for cadmium ions. The results were consistent at a certain concentration of the heavy metal ions, and exceeding the concentration resulted in complete filling of the active sites present on the clay′s surface, thus no further adsorption was observed. Additionally, the use of zeolitic volcanic tuff exhibited high efficiencies in the simultaneous removal of heavy metal ions, which had a higher selectivity towards lead ions that were present in the solution [86]. Despite their high efficiencies, naturally occurring clay and zeolitic adsorbents are often limited by selectivity challenges and low adsorption capacities, which can be improved through modification techniques. However, they result in additional challenges associated with costs and, in other cases, structural deformations blocking the active sites [21]. These modification processes improve the adsorbent′s structural properties that enhance their adsorption efficiencies. Aldhafeeri reported that the development of clinoptilolite‐chitosan composites enhanced the surface area of clinoptilolite from 23.8 m^2^/g to 30.0 m^2^/g which provided maximum adsorption capacities of 0.62 mg/g for Cu(II), 0.55 mg/g for Ni(II), and 0.40 mg/g for Mn(II) [87].

Conventional activated‐carbon (AC) adsorbents have proven effective at removing heavy metal ions from water and wastewater, outperforming clay and zeolitic adsorbents because of their microporous structure, which results in high porosity and large internal surface area [88]. They are mainly produced from any carbon‐rich material, but coal is commonly used in industrial applications that require high investment costs for large‐scale production [89]. Environmental awareness has led to the concept of using waste materials as unconventional precursors for producing efficient activated carbons [89]. Synthesizing AC from waste materials offers advantages in cost, regeneration, and sustainability [90]. Waste materials used to produce activated carbon are often readily available, have high carbon content and low ash content, and are nontoxic [90]. These waste‐derived activated carbons subsequently demonstrate higher removal efficiencies for heavy metal ions, comparable to those derived from coal. For example, tire‐derived activated carbon showed a greater ability to adsorb Pb(II), Cu(II), and Zn(II) ions from wastewater, with adsorption capacities reaching 322.5, 185.2, and 71.9 mg/g for Pb(II), Cu(II), and Zn(II) ions, respectively [89]. In contrast, commercial activated carbon exhibited capacities as low as 42.5, 15.0, and 14.0 mg/g for Pb(II), Cu(II), and Zn(II) ions, respectively. This indicates that waste‐derived activated carbons have higher removal efficiencies compared to those sourced commercially.

There has been significant advancement in heavy metal remediation employing adsorption technology, associated with the development of newer adsorbents that overcome the limitations of conventional adsorbents [91]. These materials possess higher surface areas, high porosity, tunable surfaces, increased selectivity, and regeneration potential, resulting in easier recovery that minimizes secondary pollution and higher extraction capacities [92]. These superior characteristics have made these newer materials potential candidates for the elimination of heavy metal ions from effluents and in industrial applications. For instance, coprecipitation prepared mercaptocarboxylic acid intercalated layered double hydroxides exhibited stronger selectivity towards Hg(II) ions, achieving an extraction efficiency > 99% in a solution that also contained Pb(II), Cu(II), Ni(II), and Zn(II) ions.

Metal organic frameworks are a new class of adsorbents made up of chemical functionalities with a high coordination ability towards heavy metal ions [93]. Their high porosity and large surface area report higher removal efficiencies of 97.6, 99.5, and 99.5% in the extraction of Cd(II), Cr(VI), and Pb(II) ions from wastewater using a copper‐based MOF [93]. Apart from exhibiting higher extraction capacities, MOFs are engineered for the selective extraction of heavy metal ions. For instance, even in the presence of other coexisting ions (Mg(II), Ca(II), Zn(II), Ni(II), Co(II), and Cu(II)) in solution, AMO‐MOF exhibited a higher selectivity and binding affinity (90%) towards lead ions [94].Furthermore, the modification of MOFs provides higher extraction efficiencies, rapid kinetics, and drives the selectivity towards particular heavy metal ions in solution. An example is Fe_3_O_4_‐Zr MOF, which was modified by immobilizing tripeptide glutathione providing a highly efficient adsorbent that was capable of extracting 95%–99% of Hg(II), Cd(II), and Pb(II) ions in solution after only 20 min [95].

Nanosized metal particles have also been reported as potential adsorbents for heavy metal ions owing to their large surface area that provides more binding sites. Additionally, they have tunable surfaces that can easily be modified by various functionalities. Iron oxide nanoparticles are among the most studied nanosorbents, providing higher metal ion extraction efficiencies and magnetic recovery [96]. The maximum adsorption capacities presented in Table 5 demonstrate the differences between conventional and modern adsorbents in the removal of heavy metal ions. Conventional adsorbents such as modified zeolites and activated carbon provide moderate extraction efficiencies, although agricultural‐derived activated carbon reported high extraction capacities for Pb(II) ions from synthetic solutions (322.28 mg/g). In comparison, modern adsorbents particularly metal organic frameworks and metal oxide nanoparticles provide higher extraction capabilities. Overall, the data demonstrate that modern adsorbents outperform conventional adsorbents in both the extraction and selective efficiencies. However, economic considerations need to be taken into account while employing modern adsorbents, as well as the regeneration potential and environmental sustainability.

**Table 5:** Adsorbents utilized in the removal of heavy metal ions.

Classification	Type	Adsorbent	Heavy metal ion	Optimized conditions	*Q* *m* *a* *x* (mg/g)	Ref
Conventional	Zeolite	Clinoptilolite–chitosan composites	Cu(II)	pH 6	0.62	[87]
			Ni(II)	8 g/75 mL	0.55	
			Mn(II)	2 h	0.40	
	Activated carbon	Commercial activated carbon	Pb(II)	pH 5	34.715	[97]
			Cu(II)	5 g/L	8.818	
			Zn(II)	25 mg/L	4.399	
			Cd(II)	25°C	10.816	
			Cr(VI)		26.635	
		Mahogany‐derived activated carbon	Pb(II)	pH 4.5	322.28	[98]
				3.0 g/L		
				200 mg/L		
				420 min		
				25°C		

Modern	Metal organic frameworks	3D Zn MOF [Zn_2_(DPTTZ) (OBA)_2_]	Pb(II)	pH 5 for Pb(II)	1450	[99]
			Hg(II)	pH 4 for Hg(II)	900	
				5 mg		
				100 mg/L		
				3 min		
		Copper diphenylamine metal‐organic framework	Cd(II)	pH 6	1.33	[93]
			Pb(II)	pH 2 for Cr(VI)	2.19	
			Cr(VI)	1.5 g	3.21	
				20 mg/L		
				60 min		
				25°C		
	Nanoparticles	Green synthesized iron oxide nanoparticles	Cd(II)	pH 6	105.932	[100]
				120 min		
				100 mg/L		
				1.0 g/L		
			Pb(II)	pH 5	118.764	
				90 min		
				120 mg/L		
				1.0 g/L		
		Polyethyleneimine ‐functionalized Fe_3_O_4_ magnetic nanoparticles	Pb(II)	pH 5	60.98	[101]
				1.0 g/L		
				50 mg/L		
				120 min		
				25°C		

Although adsorption is regarded as the most effective technique for heavy metal ion removal, the production of suitable adsorbent materials can be costly, and certain materials cannot be regenerated after use, limiting their application in large‐scale settings [83]. A great deal of recent work has focused on the use of adsorbents with metal‐binding capacities capable of eliminating unwanted heavy metal ions from contaminated water at a low cost. Among the adsorbents, biosorbents stand out as promising alternatives because of their low cost, high efficiency, and eco‐compatibility, resulting in the aspect of biosorption [3].

Biosorption is a biological remediation technique that utilizes specific biomasses or biomolecules to remove contaminants, such as heavy metal ions, from polluted water sources [102]. These biomasses used as biosorbents for heavy metal ions include microbial biota such as algae, fungi, and bacteria, as well as plant materials [103]. Recent research has reported on the use of biosorbents derived from agricultural wastes or industrial by‐products because of their low costs compared with the conventional natural adsorbents [37]. These biosorbents can be utilized in their natural or modified forms for pollutant removal; however, modification enhances their surface properties and improves the sorption capacity. The pollutant binding ability of these biosorbents depends on several factors, including solution pH, functional groups present, contaminant concentration, and binding characteristics [104].

The main mechanisms involved in biosorption vary depending on various factors and include ion exchange, coordination, complexation, chelation, micro precipitation, and diffusion across cell walls and membranes [105]. Many biomass‐based adsorbents from agricultural sources have been used as low‐cost options to remove heavy metal ions, showing promising results [106]. For example, Tokay and Akpinar compared the removal of copper, nickel, and zinc ions from underground water using rice husk, coconut coir, and moringa seeds. They found that moringa seeds had the highest adsorption capacities but released sulfur at acidic pH levels [107]. Additionally, sugarcane bagasse used as a biosorbent to remove lead and nickel ions from untreated refinery effluent achieved removal efficiencies of 89.31% for lead and 96.33% for nickel [106]. Agriculturally based biosorbents contain metal‐binding functional groups and are highly porous, enabling them to easily trap heavy metal ions within their pores [108]. Fruit wastes can be utilized as excellent agricultural‐derived biosorbents for water purification, offering sustainability in the sorption process.

## 5. Fruit Peel Wastes as Biosorbents

Fruits protect the body against various chronic illnesses, provide essential nutrients needed in the body, and boost the immune system; thus, they are considered a critical part of human physical health [109]. The increased health awareness on dietary habits and population growth has led to a rise in demand for fruits [109]. The most widely produced and consumed fruits are oranges, bananas, mangoes, apples, grapes, and watermelons [110]. Therefore, they contribute significantly to the amount of waste generated in households, marketplaces, and fruit processing industries [37]. The industrial processing of fruits generates waste aggregates compared with other food processing sectors, consisting of nearly 50% peels, seeds, skins, pods, shells, and pomace [109, 111]. Although these wastes are organic in nature and can biodegrade when disposed of, they slowly decompose, resulting in a massive accumulation of wastes that pollute the environment [38]. Additionally, they release greenhouse gases such as methane during decomposition that have the potential to cause global warming [38].

Reliance on conventional management practices such as landfilling and incineration has resulted in reduced land availability for waste disposal and environmental concerns such as air pollution and uncontrolled methane release, causing climatic effects [112]. Additionally, the higher moisture content of the waste results in the production of harmful gases during incineration, whereas landfilling results in eutrophication [113]. Effective utilization of this fruit waste reduces the carbon footprint and greenhouse gas emissions, thus achieving Sustainable Development Goals [35]. Biodegradability, cost‐effectiveness, durability, and lighter weight are some of the properties of fruit wastes that make them ideal for valorization [110]. The fruit waste contains a variety of bioactive compounds that can be extracted and used as value‐added products in various industrial applications, following the circular economy concept [35]. For example, pectin, a heteropolysaccharide extracted from tropical fruit peel wastes such as orange peels, is commercially acceptable as a thickening agent, especially in jellies and jams [114].

Furthermore, fruit peels, which constitute the major part of the waste, can be valorized and utilized for a variety of applications [113, 115, 116]. They are considered specialized residues because of their high content of compounds like phenols, pectin, tannins, and phytochemicals compared with those isolated from the rest of the fruit waste [109]. These compounds possess active functional groups that act as binding sites for a variety of pollutants in wastewater. Additionally, they possess a large surface area for adsorption and rough surfaces that contain these pollutants. As shown in Table 6, fruit peels have been employed as adsorbents for pollutants in wastewater, exhibiting higher to moderate removal efficiencies. The pollutant removal rate is dependent on the type of the fruit peel employed [117]. For instance, banana peels demonstrated a higher affinity for fluoride ions compared with the orange and lemon peels. This is explained by the differences in the chemical compositions of these peel biosorbents. Furthermore, banana peels are widely reported in pollutant removal from wastewater compared with the other peels. The potential application of these fruit peel wastes in the removal of a variety of contaminants provides an avenue of reviewing their use as biosorbents for heavy metal ions in wastewater. Additionally, fruit peel extracts are easily utilized as synthesis agents for nanoparticles utilized for the remediation of wastewaters contaminated with heavy metal ions.

**Table 6:** Fruit peels as biosorbents for pollutants.

Fruit peels	Target pollutants	Efficiency (%)	Ref
Banana peels	Fluoride	79.8	[117]
Orange peels	Fluoride	39.19	
Lemon peels	Fluoride	48.36	
Orange peels	Aflatoxins	90	[118]
Pomegranate peels	Cationic dyes	98	[119]
Banana peels	Anionic dyes	90	
Banana peels	Organic pollutants	91–97	[120]

### 5.1. Fruit Peel Wastes as Modifying Agents

Fruit peel waste is an abundant and environmentally friendly resource for the synthesis of nanoparticles using their extracts, offering sustainable alternatives to chemical and physical methods [121]. These extracts contain a variety of bioactive compounds that help reduce metal salts and stabilize nanoparticles by capping them, thereby providing highly efficient nanomaterials [121]. This is because they contain large quantities of phytochemicals such as flavonoids, phenolics, and alkaloids, which are reducing agents, along with functional groups that coat the nanoparticles, ensuring stability [122]. For instance, the phytochemicals in the orange peel extract reduced Au(III) to Au(0) in the synthesis of gold nanoparticles and functionalized the surface with –OH, ‐COOH, ‐CH, C=O, C=C, and C‐O groups, which increased their stability and provided binding sites for heavy metal ions [123]. The orange peel extract also reduced Ag(I) to Ag(0) in basic conditions, and the coating with the functional groups provided highly stable silver nanoparticles [124]. In addition to providing binding sites and stability, the synthesis of iron oxide nanoparticles using yali pear extracts provided a relatively rough porous surface that was occupied by Cr(VI) ions [125].

More importantly, the amount of phenolic compounds plays a role in the final structures and sizes of synthesized nanoparticles [126]. The phytochemicals can result in different‐sized and nonuniform nanoparticles, which is an indication of complicated nucleation and growth during the synthesis process [127]. For instance, in the synthesis of iron nanoparticles using orange and pomelo peel extracts, pomelo peel extract‐derived nanoparticles displayed a more even distribution compared with the nanoparticles derived from the orange peel extracts [126]. Additionally, the nanoparticles synthesized from the orange peel extracts exhibited slight agglomeration attributed to the high phenolic content of orange peel extracts (5.7) compared to that of the pomelo peel extracts (1.9) [126]. To promote highly stable nanoparticles during the synthesis process, processes such as microwave irradiation can be employed [128]. These metal nanoparticles synthesized using fruit peel extracts have been used for various applications [129–134], but very few studies have explored their potential for heavy metal ion elimination. For example, Murcott mandarin peel (*Citrus reticulata* Blanco × *Citrus sinensis* (L.) Obs.) was utilized as a precursor for the synthesis of silver nanoparticles (Ag‐NPs), in which the extracted bioactive compounds acted as both reducing and stabilizing agents during nanoparticle formation [135]. The resulting Ag‐NPs were then employed for the removal of several metal cations from industrial wastewater, which demonstrated exceptional efficiency, particularly for lead ions at the rate of 42.7 mg/g. Moreover, the solid residue obtained while preparing the extract showed promising lead ion uptake, reaching a maximum capacity of 6.6 mg/g. The synthesis of iron nanoparticles using lemon peel extracts also proved effective in the efficient elimination of Cr(VI) ions, achieving over 90% extraction efficiencies at a pH of 2, demonstrating effective applications in acidic environments [127].

The preparation of composites with green‐synthesized nanoparticles has also proven effective for eliminating heavy metal ions from wastewater. This is attributed to the additional surface area that provides more binding sites for metal ion chelation. Incorporating Fe/Pb nanoparticles synthesized using pomegranate peel extracts onto sand particles significantly increased the surface area from 0.56233 m^2^/g (sand particles) to 6.0488 m^2^/g (composite) [136]. Additionally, the pore volume increased from 0.003829 cm^3^/g to 0.008355 cm^3^/g after coating the sand particles with the nanoparticles [136]. This increase in the surface area and porosity provided higher extraction efficiencies (99%) of Cu(II) ions from aqueous solutions and a higher adsorption capacity (9.8 mg/g) compared with other reported clay‐supported materials [136].

Although fruits are a major component of plants, there is a gap in the utilization of their peel extract as a synthesis agent for nanoparticles for their application in heavy metal ion sorption. Their bioactive composition, which richly contains various functional groups that coat the nanoparticles, acts as binding sites for heavy metal ions, enhancing their removal from contaminated sources.

### 5.2. Fruit Peel Waste as Biosorbents for Heavy Metal Ions Removal

The use of fruit peel wastes as potential adsorbents for noxious metal ions has gained momentum in the recent past. This is due to their abundant availability, simple processing, unique chemical composition, renewable nature, and the fact that they do not generate secondary pollutants during treatment [137, 138]. Fruit peels are a rich source of organic macromolecules comprising carbohydrates as the major fraction, followed by proteins, lipids, minerals, and other bioactive constituents such as polyphenols and flavonoids [139, 140]. The high carbohydrate composition highlights their lignocellulosic nature and suitability as renewable biosorbent materials [141]. Comparative proximate analysis of citrus fruit residues showed that orange peels contained a higher total carbohydrate content (74.53%) than lemon peel (69.15%), whereas both exhibited comparable protein levels (7.13% and 7.81%, respectively) [139]. The carbohydrate fraction represents the combined contribution of structural and nonstructural polysaccharides, including cellulose, hemicellulose, pectin, and soluble sugars, which determine the chemical functionality of fruit peel biomass [142]. This lignocellulosic composition is associated with a variety of oxygenated functional groups, including hydroxyl, carboxyl, carbonyl, and phenolic groups that interact and bind with heavy metal ions in wastewater through various mechanisms [138, 141].

Cellulose possesses hydroxyl groups bonded by hydrogen bonds to hemicellulose, resulting in a structure with a highly reactive surface area that can adsorb metal cations [143]. Thus, the higher the cellulose content in the fruit peel waste, the higher the adsorption capacity. For instance, orange peels are reported to have a high removal efficiency for heavy metal ions because of their high cellulose content [144]. The abundant availability of cellulose in banana peels, which ranges from 12% to 59%, depending on various factors, also makes it a good biosorbent for heavy metal ions [145]. Pectin, which is abundant in the peels of citrus fruits such as oranges and lemons, mangoes, and apples, possesses carboxyl and ester groups that allow for heavy metal ion chelation [146]. Pomelo peels are reported to contain 16%–17% of pectin composed of a methylated ester part that can easily be converted to carboxylic acid groups through saponification providing anionic exchange sites for As(V) ions [147]. Lignin, a phenolic polymer, mainly contains aromatic and hydroxyl functional groups that allow for the biosorption of heavy metal ions [148].

Recent reports have demonstrated that fruit peel wastes have varying lignocellulosic compositions (Table 7). Orange peels are reported to contain 23.10% cellulose, 8.42% hemicellulose, 24.25% pectin, and 3.86% lignin [149], whereas citrus peels contained 18.84% cellulose, 17.01% hemicellulose, 23.10% pectin, and 9.16% lignin [153]. The relatively high pectin percentages in these citrus‐derived peels enhance their potential for the extraction of heavy metal ions resulting from pectin carboxyl groups that provide negatively charged sites for biosorption. Similarly, mango peels display a high pectin content (32.28%), suggesting strong ion‐exchange capacity despite their lower cellulose and hemicellulose concentrations [152]. In contrast, some fruit peels exhibit higher cellulose and lignin fractions, which influence adsorption through hydroxyl and phenolic interactions. Watermelon rind, for example, contains 50.30% cellulose, providing a high density of hydroxyl groups, although its lower pectin and lignin contents may limit direct metal complexation compared with citrus‐based materials [156]. Lastly, apple pomace, with a higher lignin content (21.49%) together with cellulose (19.79%), hemicellulose (17.30%), and pectin (12.23%), may exhibit enhanced adsorption through combined hydroxyl, phenolic, and carboxyl interactions [150].

**Table Table7:** Lignocellulosic composition of various fruit peels.

Fruit peel	Cellulose (%)	Hemicellulose (%)	Pectin (%)	Lignin (%)	Ref
Orange peels	23.1	8.42	24.25	3.86	[149]
Apple pomace	19.79	17.30	12.23	21.49	[150]
Jackfruit rind	28.56	22.57	—	2.46	[151]
Banana peel	22.12	21.35	—	14.23	
Pineapple peel	26.18	28.02	—	6.42	
Mango peel	12.03	3.66	32.28	5.39	[152]
Citrus peel	18.84	17.01	23.1	9.16	[153]
Pomegranate peels	11.0	11.6	—	12.2	[154]
Watermelon rind	50.30	4.70	—	3.50	[155]

Apart from the lignocellulosic content, the physicochemical properties of fruit peel wastes greatly influence the nature of the biosorption process and the biosorption performance. For instance, the analysis of the pH of various fruit peel powders revealed that avocado peels had a higher pH of 6.23 relative to that of yellow passion fruit peel (4.46), orange peels (4.30), and pineapple peels (4.24) [114]. These lower pH values of the pineapple, orange, and yellow passion fruit peels make them efficient biosorbents in acidic environments whereas avocado peels can effectively eliminate heavy metal ions in neutral environments [114]. In general, the utilization of fruit peel waste as biosorbents can be achieved in its natural raw form or through further treatment or modification to enhance its properties and active sites [141].

### 5.3. Biosorption of Heavy Metal Ions Using Raw Fruit Peel Wastes

The biosorptive use of fruit peel wastes in their raw form involves pretreatment stages that include washing, drying, grinding, or crushing the peel wastes to enhance their surface area, porosity, and adsorption capacities [157]. The use of fruit peel waste in this raw form is efficient, as it simplifies the pretreatment stages and saves on costs [141], but results in low adsorption capacities due to low porosity and limited binding sites, limiting removal in highly concentrated solutions. For instance raw pomegranate peels demonstrated a 25.46% extraction efficiency for Cr(VI) ions which was very low compared with the acid modified pomegranate peels that showed a 96.10% removal efficiency [158]. This was attributed to the smooth, homogenous surface of the raw pomegranate peels that contained fewer pores and voids for the adsorption of the metal ions [158]. The biosorption process using raw fruit peel wastes primarily occurs through several mechanisms, including electrostatic attraction between the negatively charged functional groups and the heavy metal ions, ion exchange between the naturally bound ions on the peels and the heavy metal ions, and chelation that involves the coordination between the functional groups present on the peel and the heavy metal ions [36].

The structural characteristics and chemical composition of fruit peels make them efficient biosorbents for heavy metal ions in wastewater. The FTIR analysis of the various functional groups responsible for the extraction of heavy metal ions showed that orange peels contain –OH, C‐H, C=O, C=C, N‐H, and C‐OH groups [159]. These groups provided active sites for the removal of Cu(II) and Pb(II) ions from synthetic solutions, revealed from the shift of the functional groups peaks in the FTIR spectra [159]. This shift generally reveals the chemical interaction of the heavy metal ions with the functional groups as displayed by the shift in the alkyl groups from 803 cm^−1^ before the adsorption of Cu(II) and Pb(II) in a binary system using banana peels to 906 cm^−1^ with an increase in steepness [160]. Additionally, physical properties revealed by the BET analysis showed that banana peels have a larger surface area and total pore volume (73.2 m^2^/g and 0.231 cm^3^/g) relative to that of orange peels (63.2 m^2^/g and 0.195 cm^3^/g) and of pomegranate peels (35.9 m^2^/g and 0.109 cm^3^/g) [119]. This shows that banana peels are likely to display higher extraction rates of heavy metal ions compared with orange or pomegranate peels.

Various types of raw fruit peel wastes have been utilized as potential biosorbents for heavy metal ions, as reported in recently published works. For instance, orange peels, being rich in cellulose, lignin, and pectin, have demonstrated a high affinity for cadmium, nickel, lead, and chromium ions [137, 161, 162]. Akinhanmi et al. [137] reported an adsorption capacity as high as 128 mg/g for Cd(II) ions at the optimized conditions from synthetic aqueous solutions utilizing orange peel powder. Orange peel powder also demonstrated almost 100% removal efficiency for lead ions at the optimum metal ion concentration of 20 mg/L [162]. Similarly, the comparison of banana and orange peels for the removal of cadmium, nickel, and chromium ions from landfill leachate showed that the orange peels had the highest potential in eliminating the metal ions [161]. The better performance of the orange peels was attributed to their active functional groups, which provided sites for adsorption, and the large surface areas revealed by the SEM characterization results. The removal efficiency for the metal ions ranged from 99.90% to 99.99% for nickel, 97.00% to 99.98% for cadmium, and 59.30% to 98.18% for chromium for the orange peel adsorbent, and 99.55% to 99.99% for Ni, 99.16% to 99.96% for Cd, and 45.67% to 82.91% for Cr for the banana peel adsorbents.

In another related study, BET analysis of orange peel powder revealed a large surface area that provided more active sites for adsorption, resulting in the highest removal efficiency of 99.2% [163]. Similarly, the utilization of banana, lemon, mango, and orange peels proved efficient for the removal of Pb(II), Cd(II), and Co(II) ions from water [55]. From the findings of this study, lemon and orange peels had the highest removal efficiencies because of their rugged rough surfaces, large pores, and active functional groups favorable for adsorption, whereas Pb(II) ions exhibited the highest removal, followed by Cd(II) and Co(II) ions because of their high electronegativity [164]. Dragon fruit peels were also found to be effective biosorbents for Co(II), Cd(II), Pb(II), and Ni(II) ions from single and multimetal solutions, with a selectivity towards Pb(II) ions [165].

For the efficient biosorptive removal of heavy metal ions, various factors including pH, metal ion concentration, adsorbent dose, temperature, interaction time, and particle sizes are considered. The point of zero charge (pHpzc) influences the heavy metal ions biosorption process as it provides the pH that describes the neutral point of an adsorbent. Different peel wastes exhibit different pHpzc resulting in different ideal pH values for maximum extraction efficiencies. For instance, while examining banana and orange peels for the removal of Cu(II) and Pb(II) metal ions from binary solutions, orange peels displayed a pHpzc value of 3.85 whereas banana peels had a slightly higher pHpzc value of 4.83 [159, 160]. This difference in the pHpzc value makes orange peels highly efficient at eliminating the heavy metal ions at a lower pH threshold (acidic wastewater) compared with the banana peels [119]. This is because at lower pH ranges (pH 4 and 5) the surface of the orange peel adsorbents becomes negatively charged and can efficiently attract the heavy metal ions [119].

The summarized studies in Table 8 demonstrate that raw fruit peel biosorbents potentially extract heavy metal ions from solutions. Among the reported biosorbents, orange peels consistently demonstrated higher extraction efficiencies for Cu(II), Pb(II), Cd(II), La(III), and Y(III) ions for both binary and single metal systems. Banana peels also showed excellent Pb(II) removal, whereas dragon fruit and pomelo peels displayed greater selectivity, particularly for Pb(II), with dragon fruit peel exhibiting the highest adsorption capacity (82.64 mg/g) among the fruit peel biosorbents presented. Although raw fruit peel wastes have exhibited remarkable sorption capacities for heavy metal ions, it is highly dependent on the peel structural properties, the type of metal ion, and the operating process conditions. However, they have demonstrated limited adsorption, especially in high concentrations of the ions attributed to their smooth surfaces that reduce the number of active sites available for the binding of contaminants [157, 169]. Nevertheless, their adsorption capacities can be increased by increasing their surface roughness and porosity through various modification methods.

**Table 8:** Biosorptive removal of heavy metal ions using unmodified fruit peels.

Fruit peel	Heavy metal ions	System type	Optimized conditions	Extraction efficiency (%)	Extraction capacity *Q* *m* *a* *x* (mg/g)	Ref
Orange peels	Cu(II)	Binary	100 mg/L, 1 g adsorbent dose	87.32	38.18	[159]
	Pb(II)			98.85	40.05	

Banana peels	Cu(II)	Binary	100 mg/L, pH 5, 1 g adsorbent dose, and 75 *μ*m particle size.	88.94	29.26	[160]
	Pb(II)			99.79	39.32	

Dragon fruit peel	Co(II)	Single	100 mg/L metal ion concentration, 0.1 g adsorbent dose, 24 h contact time, pH of 5.5, at 298 K	30	6.013	[165]
				63	17.95	
				77	82.64	
	Cd(II)			11	5.322	
	Pb(II)					
	Ni(II)					

Orange peels	Cu(II)	Single	10 mg/L metal ion concentration, 120 min contact time, pH 6	96.9	2.78	[166]
				98.1	2.57	
	Cd(II)					

Pomelo fruit peels	Pb(II)	Single	50 mg/L metal ion concentration, pH 5.5, 0.1 g adsorbent dose, 120 min contact time for Pb(II) ions and 100 min for Cd(II) ions at 303 K	90	47.18	[167]
				87	13.35	
	Cd(II)					

Orange peels	La(III)	Single	pH of 6, temperature of 40°C, dosage of 3 g/L, contact time of 30 min, and initial ion concentration of 32 mg/L.	95.49	37.61	[168]
	Y(III)			76.31	31.10	

### 5.4. Types of Modification of Fruit Peel Wastes

As outlined earlier, raw fruit peel biosorbents have low sorption capacities and limited selectivity for heavy metal ions. Surface modification alters the surface of these fruit peel biosorbents by adjusting their physical and chemical characteristics, thereby enhancing their biosorption efficiency [170] and directing their selectivity towards specific heavy metal ions [171]. For instance, in the presence of competing ions, amine‐modified passion fruit peel biosorbents showed the highest Cr(VI) selective removal efficiency of 99.9% [172], whereas in a binary system containing lead and copper ions, orange peel biochar showed binding selectivity towards Pb(II) ions [37]. A variety of modification methods, including physical, thermal, and chemical activation as well as composite formation, are employed for the activation of fruit peel wastes to enhance their biosorptive capacity [143].

#### 5.4.1. Chemical Modification

Chemical modification techniques involve the use of various chemicals to enhance the structural properties of fruit peel wastes and introduce desired functional groups that provide additional binding sites for heavy metal ions to improve biosorption capacities [157]. This functionalization technique primarily focuses on the removal of soluble compounds present in the wastes that could hinder the biosorption process, leading to a more porous structure [143]. Additionally, simple acid and base treatments that are commonly employed for the functionalization of fruit peel wastes make their binding sites more available to react with heavy metal ions through various mechanisms [171].

##### 5.4.1.1. Acid Modification

This wet oxidation process involves using mineral acids or other oxidants such as HNO_3_, H_2_O_2_, HClO, H_2_SO_4_, H_3_PO_4_, and HCl, along with organic acids like oxalic, carboxylic, and acetic acids [170]. Acidifying fruit peel biosorbents causes a series of physical and chemical changes that greatly affect their subsequent performance in biosorption of heavy metal ions. The first physical change observed is darkening of the fruit peel biosorbents, caused by oxidation reactions with compounds in the peels, leading to the formation of colored compounds [173]. For example, rambutan, passion, and dragon fruit peels acidified with 4 mol/L of H_2_SO_4_ for improved removal of copper, lead, and cadmium ions from water were darker than untreated peels because of these oxidative reactions [104, 174].

Acidification also changes the surface morphology of fruit peel waste, mainly affecting surface area and porosity. Surface area is a crucial factor in biosorption, representing the number of active sites on the biosorbent surface for binding heavy‐metal ions [65]. Increasing the surface area enhances the biosorbent′s removal efficiency. Acid treatment is reported to increase the surface area of fruit peel biosorbents, providing more binding sites for heavy metal ions. For example, in a previous report, the surface area of banana peel waste increased from 0.0363 to 0.0507 m^2^/g after sulfuric acid treatment, as shown by BET studies [175]. This significant increase was likely due to breaking bonds within the fruit peels, which causes lignocellulosic compounds to separate or form larger cage‐like molecules within the structure, resulting from tannins undergoing condensation and solidification [175]. Concerning porosity, acid treatment dissolves soluble compounds in the peels that might interfere with biosorption, leading to a more porous structure that provides extra sites for metal ion binding [104]. The increase in surface roughness after acidification also increases the porosity for an enhanced biosorption capacity [158].

For the chemical influence, acidification of fruit peel biosorbents increases the number of oxygen‐containing functional groups that are present on the surface while reducing the mineral content [176]. These added acidic functional groups increase the negative charge density on the surface, particularly upon deprotonation at a suitable pH, which favors the electrostatic attraction and complexation of positively charged metal ions [170]. For example, acid‐oxidized pineapple fruit peels have been shown to improve Cd(II) and Pb(II) metal ions uptake because of the introduction of carboxylic and hydroxyl groups on the biosorbent′s surface [177]. Acidified rambutan peels also showed the highest removal efficiency for Cu(II) ions, as shown in Table 9, because of the additional functional groups of carboxyl and hydroxyl that led to a greater exchange of the ions at the sorption site [104]. Acidification also chemically influences the pHpzc of the biosorbents by shifting it towards more acidic values, demonstrated by the pHpzc value of acidified pomegranate peels at 4.0 [158]. These physical and chemical changes result in an enhanced biosorption performance and stronger binding affinities compared with the raw peels. For instance, chemically activating the passion, rambutan, and dragon fruit peels with 4 mol/L of H_2_SO_4_ improved their biosorption capabilities for the removal of Cu(II), Pb(II), and Cd(II) ions from aqueous solutions, as shown by the improved removal efficiencies and enhanced adsorption capacities in Table 9.

**Table 9:** Comparison of the adsorption capacities of the untreated and chemically treated peels [104, 174].

Fruit peel	Heavy metal ion	Untreated peels		Chemically treated peels	
		Removal efficiency (%)	Adsorption capacity (mg/g)	Removal efficiency (%)	Adsorption capacity (mg/g)
Rambutan peels	Cu(II)	95.73	38.29	97.00	38.80
	Pb(II)	49.58	19.84	97.87	39.16
	Cd(II)	94.87	35.92	97.10	37.60

Dragon fruit peel	Cu(II)	53.33	21.33	55.20	22.08
	Pb(II)	76.62	30.64	92.92	33.04
	Cd(II)	91.30	33.04	91.30	38.04

Passion fruit peels	Cu(II)	52.28	20.91	58.93	23.57
	Pb(II)	84.68	33.88	94.54	37.80
	Cd(II)	87.67	30.16	93.43	34.72

Variations in the composition of fruit peels explain the differences in their biosorption performance. Following acid treatment, peels with higher concentrations of pectin, cellulose, hemicellulose, and phenolic compounds typically offer more hydroxyl and carboxyl groups that can take part in surface complexation and ion exchange [104, 175, 177]. For instance, pineapple and banana peels have shown improved biosorption of Cd(II) and Pb(II) ions after oxidation because more oxygenated functional groups are available, whereas rambutan peel has shown improved Cu(II) and Pb(II) removal because of improved ion exchange interactions following acid modification [104, 175, 177]. These results show that the original chemical structure of each fruit peel affects the efficacy of acid modification in addition to the type of treatment used. However, because reported efficiencies are affected by differences in modification conditions and biosorption parameters comparing the adsorption capacities of various fruit peel biosorbents is quite difficult.

##### 5.4.1.2. Alkali Modification

This modification strategy involves treating fruit peel waste with strong bases, such as sodium hydroxide and potassium hydroxide, to enhance the biosorbent′s ability to efficiently eliminate heavy metal ions [178]. During alkaline treatment of fruit peel wastes, hydrolysis reactions that cause the high dissolution of organic compounds, often limiting their biosorption capacity, result in considerable disintegration, forming highly effective biosorbents [179]. This is a result of the improved surface properties, such as an increase in surface area, which provides more active binding sites. For instance, modifying lemon peels with sodium hydroxide increased the surface area from 0.4090 to 0.5367 m^2^/g [180]. However, the increase in surface area is not significant, indicating that the modifying power of sodium hydroxide is weaker compared with other chemical modifiers. This increase in surface area subsequently increases the pore volume, for example, from 0.002 to 0.004 cm^3^/g when melon peel wastes were modified with sodium hydroxide, resulting in a highly porous surface for biosorption [181].

Additionally, alkali modification introduces more hydroxyl functional groups on the surface of fruit peel biosorbents that play a huge role in the efficient removal of heavy metal ions [180]. These physical and chemical changes increase the biosorption capacity of fruit peels, such as the high sorption capacities of modified melon peels exhibiting 77.76 mg/g for Cu(II) ions, 76.16 mg/g for Cd(II) ions, and 191.93 mg/g for Pb(II) ions [181]. The higher removal efficiency for Pb(II) ions was attributed to its higher electronegativity and softer acidic nature, as well as its strong interaction with the hydroxyl functional groups that were present on the surface, as explained by the Soft‐Hard Acid‐Base principle. Alkali modification also increases the selectivity of fruit peel biosorbents towards particular heavy metal ions in water. Jahin et al. [182] demonstrated that banana peel biosorbents modified with potassium hydroxide could potentially eliminate about 62% Cd(II), 6.52% Cr(II), 28.5% Fe(II), 95.83% Pb(II), 54.73% Mn(II), 14.29% Mo(II), 24.49% Se(II), and 79.80% Sr(II) ions from wastewater. The results show that the biosorbent had a high selectivity towards Pb(II) ions, because of its higher electronegativity, compared with other ions present in the wastewater and the oxygenated functional groups on the surface of the modified biosorbent that had a high affinity for them [182]. The basic environment also plays a role in the effective attraction of the positively charged metal ions through electrostatic interactions, based on the pHpzc of biosorbents and the ideal pH for efficient removal.

The subsequent use of alkali modifiers with other chemical agents further enhances the biosorption capacity of fruit peels, but results in additional costs and environmental issues because of potential leaching [141]. For instance, modifying banana, granadilla, and orange peel wastes successively with sodium hydroxide and calcium acetate solutions at optimum concentrations resulted in the solubilization of the organic compounds, increased the surface area, and provided more functional groups on the surface for the effective removal of Zn(II) ions [143]. The batch adsorption results indicated that the highest efficiency was recorded at 0.8 M NaOH and Ca(CH_3_COO)_2,_ of 92.57%, 97.13%, and 88.21% for the banana, orange, and granadilla peels, respectively. Orange peels had the highest removal efficiencies due to their greater porosity and surface area, as indicated by the SEM images.

Generally, alkali‐modified fruit peel biosorbents′ adsorption efficiency varies depending on the lignocellulosic composition, the degree of surface functionalization, and the hydroxyl groups′ affinity for various heavy metal ions. NaOH‐modified melon peel demonstrated the best adsorption capacities among the investigated studies, particularly for Pb(II) ions suggesting the significance of improved surface accessibility and hydroxyl‐mediated interactions in metal ion uptake [181]. Conversely, in multimetal wastewater, KOH‐modified banana peels showed high selectivity towards Pb(II), indicating that metal‐specific interactions also affect biosorption efficiency [182]. Similar to banana and granadilla peels, orange peels modified with NaOH/calcium acetate showed greater biosorptive uptake of Zn(II) ions [143]. This was explained by improved porosity and surface accessibility following modification.

Although chemical modification has shown great potential in enhancing the sorption capacity of fruit peel wastes, critical gaps still exist. For example, the choice of chemical modifiers and the optimal conditions for modification are often not systematic, limiting comparisons, and the issue of residual chemical leaching raises health concerns [183]. Furthermore, their application is focused on single‐metal ion systems, thereby evading the effects of competing ions and other contaminants present in wastewater samples [183]. Most of the chemical reagents employed are also synthetically manufactured, which have adverse effects on both the environment and human health [184]. This necessitates the need for the adoption of greener reagents for the modification of fruit peel wastes to align with the Sustainable Development Goals.

#### 5.4.2. Thermal Modification

The thermal modification process alters the structure of the fruit peel waste, making it more porous and increasing its surface area while also interfering with its chemical composition, either partially or completely [171]. Hydrothermal carbonization and pyrolysis are the two major thermal processes involved in the thermal improvement of fruit peel wastes.

##### 5.4.2.1. Hydrothermal Carbonization

This is a highly effective thermochemical process that converts raw fruit peels into a carbon‐rich hydrochar at moderate temperatures ranging from 180°C–280°C, using water as the reaction medium [185, 186]. The process involves the dehydration and decarboxylation of the functional groups present in the fruit peels, resulting in an increased carbon content, higher carbon density, and better biosorption performance by the highly porous hydrochar [186, 187]. For instance, in a past study, hydrothermally carbonized banana peel wastes exhibited a mesoporous structure with uneven particle size distribution and increased porosity, as shown by BET analysis results [188]. Additionally, the treatment increased the surface area of the banana peel biosorbent from 1.10 m^2^/g to 1.601 m^2^/g at 200°C, and at 300°C, it reached 9.194 m^2^/g. This demonstrated that subjecting fruit peel wastes to hydrothermal carbonization at higher temperatures significantly increases the surface area and active sites available for binding heavy metal ions. Although decarboxylation reactions remove carboxyl groups present on the peel surfaces that promote the biosorption of heavy metal ions, reactions involving the opening of the glycosidic rings in cellulose and lignin intensify other peaks, such as the carbonyl group peak, providing binding sites for toxic ions [188, 189]. These physical and chemical changes result in an enhanced removal efficiency of heavy metal ions, as demonstrated by a 24% increase in cadmium ion removal efficiency from 75% to 99% using banana peel‐derived hydrochar [188].

It has been shown that improving the surface affinity of hydrothermal carbon further enhances its biosorption performance for heavy metal ions present in wastewater. Indeed, in a study involving the surface comparison of modified and unmodified hydrothermal carbon, it was shown that the unmodified hydrothermal carbon had a smooth surface with fewer microspheres, whereas many carbon microspheres of different sizes were present on the surface of the chemically modified hydrothermal carbon, resulting in a porous surface [187]. Additionally, increasing the concentration of the phosphoric acid as the chemical modifier resulted in a rough and uneven surface, increasing the surface porosity of the hydrothermal carbon derived from banana peels [187, 190]. Furthermore, it has been demonstrated that the chemical activation of hydrothermal carbon also increases the number of oxygen‐containing functional groups present, providing more active sites for biosorption reactions [187, 191]. Similar findings were also demonstrated by acid‐treated pomegranate‐derived biochar, which exhibited a microporous and loose structure with an increased surface area and porosity, which enhanced the adsorption capacity by 26.9% [192]. Acids have been proven as better chemical modifiers than bases, illustrated by the high surface area and pore volume results obtained from acid modifications [193].

##### 5.4.2.2. Pyrolysis

This thermochemical process involves the thermal decomposition of fruit peel waste in the absence of oxygen at low temperatures over an extended heating period, resulting in higher quantities of biochar [37]. Raw fruit peel biosorbents are often limited by their high moisture content, which occupies the pores and reduces the active sites available for metal ions [194]. This issue can be addressed through pyrolysis, a process that significantly reduces moisture levels [194]. The pyrolysis temperature greatly affects the physical and chemical properties of the resulting biochar. Zhang et al. [195] showed that increasing the pyrolysis temperature decreased mango peel biochar yield from 43.2% at 300°C to 27.0% at 700°C, indicating that lower pyrolysis temperatures produce more biochar. Besides yield, the pyrolysis temperature also influences the surface properties of biochar, especially surface area and porosity. It has been reported that higher temperatures develop the surface structure by destroying organic functional groups, which increases surface area and creates micropores, thereby raising porosity [195].

Pyrolyzing fruit peel waste also chemically alters its pHpzc, which determines the surface charge of biochar. For example, in a previous report, the process changed the surface of orange peels from acidic to basic, with the pHpzc value shifting from 3.83 to 10.03, indicating the presence of basic functional groups capable of attracting and binding positively charged metal ions [37]. This change is influenced by pyrolysis temperature, as seen in the increase from 6.87 at 300°C to 9.06 at 700°C for mango peel biochar [195]. Furthermore, this thermal process reduces the functional groups on the surface of fruit peels by volatilizing and decomposing organic acids and compounds originally present [37]. However, the increased levels of carbon, oxygen, and hydrogen resulting from the decomposition of lignocellulosic components provide more active sites for the biosorption of toxic ions [194]. All these physical and chemical changes increase the removal efficiency of heavy metal ions, such as the 97% removal efficiency that was obtained by banana peel‐derived biochar after 30 min of contact with the metal ions [196].

Chemical activation of biochar derived from fruit peels further enhances their binding affinity for toxic heavy metal ions. This is as a result of an increase in the oxygen‐containing functional groups that were removed by the pyrolysis process, which serve as binding sites and an increased surface area, such as the reported 200 m^2^/g of phosphoric acid‐activated banana peels‐derived biochar that demonstrated a 94% removal efficiency for chromium ions [197]. In another report, the activation of banana peel‐derived activated carbon with ZnCl_2_ facilitated the formation of pores in the carbon structure and increased the surface area, leading to a 5% increase in the biosorption of lead ions [198]. Consequently, the integration of Fe_3_O_4_ in calcined biochar obtained from banana peels significantly increased the surface area (from 95.89 to 323.2 m^2^/g) and the pore volume (from 0.19 to 0.34 cm^3^/g), which significantly increased the extraction efficiency of Cu(II) ions (from 37.6 to 47.9 mg/g). This integrated concept provided a highly effective adsorbent for Cu(II), Zn(II), and Hg(II) ions with a magnetic property that allowed easier recovery after biosorption applications [199]. Although, further modification of activated fruit peel biochars or activated carbon results in negative implications of reducing the porosity and surface area in some reported cases [200]. Tran et al. reported that the addition of Al_2_O_3_ and chitosan to banana peel‐derived activated carbon significantly blocked the micropores that were formed by ZnCl_2_ activation, decreasing the specific surface area from 1045 m^2^/g to 19 m^2^/g [198]. This affected the biosorption performance of the formed composite.

#### 5.4.3. Metal‐Oxide Modification

This modification strategy involves treating various fruit peel forms with metal oxide‐containing compounds, which possess unique properties for heavy metal ion sorption. Several types of metal oxides, including zinc oxide, magnesium oxide, titanium dioxide, magnetite, goethite, and iron (III) oxide‐hydroxide [201], have been utilized in recent studies to improve the ability of various peel wastes in eliminating toxic metal ions. Metal‐oxide modifications alter the physical as well as the chemical characteristics of fruit peel biosorbents, resulting in newer structures with an enhanced biosorption capacity. Depending on the type of metal oxide added to raw powders or beads, fruit peel wastes display different color characteristics. For example, pomelo beads doped with zinc oxide are cream‐colored, those doped with titanium dioxide are whitish, and beads doped with both zinc oxide and titanium dioxide are light cream in color [202]. Additionally, banana powder beads, which are black, turn dark brown when doped with zinc oxide and light brown when doped with magnesium oxide [201]. Therefore, metal‐oxide modifications cause physical changes in the color of fruit peel biosorbents. Analyzing the physicochemical parameters, such as surface area, pore volume, and pore sizes, reveals that metal‐oxide modification increases surface area and pore volume while decreasing pore sizes [203]. For instance, it has previously been reported that the surface area of banana powder beads increased from 4.33 m^2^/g to 16.60 m^2^/g when doped with magnesium oxides, and the surface area of orange peels rose from 0.431 m^2^/g to 0.896 m^2^/g when doped with iron (III) oxide‐hydroxide [31, 201]. This increase in surface area provides a more active surface for the biosorption of heavy metal ions. Furthermore, the addition of metal oxides to fruit peel biosorbents provides additional binding sites for the efficient removal of heavy metal ions, as shown in Figure 2. SEM micrographs revealed the successful incorporation of nickel oxide into the orange peel matrix, which played an active role in the enhanced biosorption of lead and cadmium ions [169].

**Figure 2:**
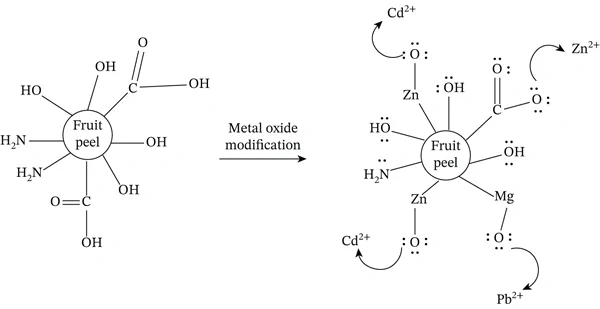
Simplified representation of the metal‐oxide modification of fruit peels.

More importantly, it has been demonstrated that metal oxide doping also chemically affects the pHpzc of the fruit peel biosorbents. For example, a higher pHpzc value of 7.19 of pomelo beaded sorbents was obtained when zinc oxide was added, compared with when titanium oxide was added, which gave a pHpzc value of 5.36 [202]. Conversely, when banana powder beads were doped with zinc oxide, they exhibited a slightly acidic pHpzc value of 6.75, which was higher than that of the banana powder beads of 5.37 [201]. These physicochemical changes resulting from metal‐oxide modifications lead to higher removal efficiencies of heavy metal ions. For instance, banana powder beads doped with magnesium oxides demonstrated 99.59% removal efficiency of cadmium ions, which was higher than the 89.18% of the banana powder beads [201]. These changes also resulted in an increase in the lead removal efficiency from 69.78% to 95.29% at the ideal conditions after iron (III) oxide‐hydroxide modification of orange peel powder [31] and removal efficiencies of almost 100% for lemon peel beads doped with iron (III) oxide‐hydroxide [203].

In another related study by Foroutan et al., [204] it was demonstrated that modifying biochar sourced from banana peel wastes using magnetite (Fe_3_O_4_) and zeolitic imidazole framework‐67 (ZIF‐67) nanoparticles significantly increased the specific surface areas from 4.70 m^2^/g to 78.83 m^2^/g and 1212.40 m^2^/g after modification using magnetite alone and utilizing both magnetite and ZIF‐67, respectively. This resulted in higher removal efficiencies of 97.52% for biochar/Fe_3_O_4_ and 99.14% for the biochar/Fe_3_O_4_/ZIF‐67 with maximum adsorption capacities of 30.33 mg/g and 50.78 mg/g, respectively. Neisan and colleagues [205] also revealed that the modification of activated carbon derived from orange peel wastes using titanium dioxide nanoparticles increased the surface area of the activated carbon from 2.40 to 6.06 m^2^/g, resulting in higher removal efficiencies of 99.90% of copper ions from synthetic water samples.

Although the metal‐oxide modification process enhances the biosorption performance of fruit peel forms towards heavy metal ions, it possesses several critical challenges and gaps. They include the lack of standardized modification strategies limiting cross‐comparisons of studies, potential leaching of metal oxides, which causes secondary pollution, their limited applicability in wastewater samples to assess the scalability, and the fate of spent adsorbents, including safe disposal or the recovery of the heavy metal ions or the metal oxides, is not addressed. Thus, future studies should address these challenges and focus on the life‐cycle assessment of the adsorbents to ensure environmental protection.

#### 5.4.4. Comparative Evaluation of the Modification Strategies

The reported studies show that by changing the surface morphology, porosity, functional groups, and surface charge of fruit peel wastes, chemical, thermal, and metal‐oxide modifications significantly enhance their biosorption performance. These modification strategies improve the biosorption performance of these fruit peel biosorbents through various mechanisms. Chemical modification mainly introduces additional oxygen‐containing functional groups that bind heavy metal ions through ion exchange, electrostatic attraction, and surface complexation [175, 181, 182]. Thermal modification, on the other hand, increases the carbon content, surface area, and porosity, creating more sites for metal ion uptake [187, 195, 197]. However, excessive thermal treatment reduces the functional groups involved in biosorption [198]. Furthermore, metal‐oxide modification increases the surface area, modifies the surface charge, and introduces additional binding sites, allowing stronger interactions with heavy metal ions through surface complexation and electrostatic attraction [31, 201, 202]. Therefore, chemical modification mainly improves surface chemical functionality; thermal modification enhances structural properties; and metal‐oxide modification combines both surface and chemical improvements, resulting in the highest reported biosorption efficiencies.

Although metal oxide modified biosorbents demonstrate higher biosorptive performance, chemical modification techniques are advantageous from an economic and practical standpoint. This is because of the straightforward procedures involved during modification. However, their sustainability is impacted by the use of synthetic reagents, possible chemical leaching, and additional treatment needs. Although metal‐oxide modification requires extra modification steps and modifying agents that may increase complexity, thermal modification produces stable carbonaceous materials but requires higher energy inputs. Additionally, reported biosorption performances are impacted by differences in modification conditions, biomass composition, and biosorption parameters. Thus, direct comparison between studies is challenging, limiting reproducibility. Majority of the research has also been done in controlled laboratory settings, especially for single‐metal systems, restricting performance evaluation in wastewater systems.

##### 5.4.4.1. Chemical Modification

Chemical functionalization significantly improves the adsorption performance of fruit peel biosorbents, as displayed in Table 10. However, the effect on the adsorption performance is dependent on the chemical modifier and the target heavy metal ion. Among the summarized chemically modified biosorbents, amine‐modified passion fruit peels exhibited the highest adsorption capacity of 675 mg/g for Cr(VI) at pH 2. This indicated the effectiveness of amine groups in enhancing chromium adsorption under acidic conditions. Acid activation using H₂SO₄ also improved the extraction efficiency of Cu(II) ions. H₂SO₄‐activated rambutan peels achieved the highest Cu(II) adsorption capacity (192.31 mg/g), followed by passion fruit peels (121.95 mg/g) and dragon fruit peels (92.59 mg/g). The improved performance was attributed to acid treatment, which removes impurities, increases surface roughness and porosity, and exposes additional oxygen‐containing functional groups that act as adsorption sites [104]. In comparison, NaOH–Ca(CH₃COO)₂‐modified fruit peels exhibited lower adsorption capacities for Zn(II) ions, ranging from 16 to 27 mg/g. Among the reported NaOH–Ca(CH₃COO)₂‐modified biosorbents, modified orange peels showed the highest Zn(II) adsorption capacity, followed by banana peels and granadilla peels. Generally, although alkaline treatment enhanced the accessibility of surface functional groups, the adsorption performance was lower than that of acid and amine modifications.

**Table 10:** Comparison of chemically functionalized fruit peel biosorbents.

Biosorbents	Type	Heavy metal ions	Optimum conditions			Adsorption capacity *Q* *m* *a* *x* (mg/g)	References
			Temp (°C)	pH	Dose (g)		
Amine‐modified passion fruit peels	Single	Cr(VI)	25	2	0.1	675	[172]
H_2_SO_4_‐activated rambutan peels	Single	Cu(II)	30	4	0.25	192.308	[104]
H_2_SO_4_‐activated passion fruit peels	Single	Cu(II)	30	4	0.25	121.951	
H_2_SO_4_‐activated dragon fruit peels	Single	Cu(II)	30	5	0.25	92.593	
NaOH‐Ca(CH_3_COO)_2_ modified banana peels	Single	Zn(II)	25	5	1	25.59	[143]
NaOH‐Ca(CH_3_COO)_2_ granadilla peels	Single	Zn(II)	25	5	1	16.61	
NaOH‐Ca(CH_3_COO)_2_ orange peels	Single	Zn(II)	25	5	1	27.48	

##### 5.4.4.2. Metal‐Oxide Functionalization

Metal‐oxide functionalization enhances the adsorption capacity of fruit peel biosorbents, particularly for Cd(II) and Pb(II) ions, as demonstrated in Table 11. Among the summarized biosorbents, magnesium oxide‐doped banana powder beads exhibited the highest adsorption capacity for Cd(II), reaching 454.55 mg/g, followed by zinc and magnesium oxide‐doped beads (303.03 mg/g) and zinc oxide‐doped beads (232.56 mg/g). These findings indicate that the type of metal oxide added influences the adsorption performance, with magnesium oxide providing more effective adsorption sites for Cd(II). Orange peels functionalized with nickel oxide nanoparticles and alginate also demonstrated high adsorption capacities for both Cd(II) and Pb(II). Under single‐metal conditions, adsorption capacities of 138.25 mg/g for Cd(II) and 276.00 mg/g for Pb(II) were reported. Even higher adsorption capacities were observed under binary‐metal systems, reaching 224.00 mg/g for Cd(II) and 317.10 mg/g for Pb(II), indicating that this composite biosorbent performs effectively in multimetal wastewater systems. In contrast, iron (III) oxide‐hydroxide‐doped orange peels exhibited a much lower adsorption capacity (2.303 mg/g) for Pb(II). This lower performance may be attributed to differences in the modifying agent, preparation method, or experimental conditions, highlighting that not all metal‐oxide modifications produce comparable improvements in biosorption. Overall, these findings indicate that metal‐oxide functionalization is highly effective in improving the adsorption performance of fruit peel biosorbents by improving the surface properties. However, the extraction efficiency depends on the type of metal oxide employed and the target heavy metal ion.

**Table 11:** Comparison of metal oxide‐functionalized fruit peel biosorbents.

Biosorbent	Type	Heavy metal ions	Optimum Conditions			Adsorption capacity *Q* *m* *a* *x* (mg/g)	References
			Temp (°C)	pH	Dose (g)		
Banana powder beads doped with zinc oxide	Single	Cd(II)	25	5	0.08	232.56	[201]
Banana powder beads doped with magnesium oxide	Single	Cd(II)	25	5	0.04	454.55	
Banana powder beads doped with zinc and magnesium oxide	Single	Cd(II)	35	5	0.06	303.03	
Orange peels infused with nickel oxide nanoparticles and alginate	Single	Cd(II)	25	5.5	0.25	Single	[169]
		Pb(II)				138.25	
						276.00	
	Binary					Binary	
						224.00	
						317.10	
Orange peels doped with iron (III) oxide‐hydroxide	Single	Pb(II)	25	5	3	2.303	[31]

##### 5.4.4.3. Thermal Modification

Thermally modified biosorbents displayed moderate adsorption capacities (Table 12), generally below 60 mg/g, with improved performance observed when combined with surface coatings such as Al₂O₃–chitosan. Among the hydrothermal carbons, KOH‐modified banana peel hydrothermal carbon achieved a slightly higher Pb(II) adsorption capacity (42.92 mg/g) than H₃PO₄‐modified banana peel hydrothermal carbon (40.64 mg/g) under similar operating conditions (25°C and pH 6). Orange peel biochar produced at 500°C also demonstrated moderate adsorption capacities, reaching 28.06 mg/g for Cu(II) and 30.12 mg/g for Pb(II). Although the reported capacities were lower compared with those reported for chemically and metal oxide‐functionalized biosorbents, they demonstrated that thermal treatment alone can produce effective adsorbents for heavy metal ion removal. Further enhancement was achieved by combining thermal modification with surface coatings. H₂SO₄‐activated banana peel carbon coated with Al₂O₃–chitosan exhibited the highest adsorption capacities among the thermally modified biosorbents, with 57.10 mg/g for Pb(II) and 46.90 mg/g for Cd(II). In comparison, ZnCl₂‐activated banana peel carbon coated with Al₂O₃–chitosan adsorbed 39.0 mg/g of Pb(II). These findings demonstrate that incorporating surface coatings provides additional binding sites, leading to improved adsorption performance.

**Table 12:** Comparison of thermally functionalized fruit peel biosorbents.

Biosorbent	Type	Heavy metal ions	Optimum Conditions			Adsorption capacity *Q* *m* *a* *x* (mg/g)	References
			Temp (°C)	pH	Dose (g)		
KOH‐modified banana peel hydrothermal carbon	Single	Pb(II)	25	6	0.1	42.92	[191]
H_3_PO_4_‐modified banana peel hydrothermal carbon	Single	Pb(II)	25	6	0.2	40.64	[187]
Orange peel biochar at 500°C	Single	Cu(II)	25	5	0.1	28.06	
	Single	Pb(II)				30.12	
Banana peel H_2_SO_4_ activated carbon coated with Al_2_O_3_–chitosan	Single	Pb(II)	25	6	0.1	57.1	[193]
		Cd(II)				46.9	
Banana peel ZnCl_2_ activated carbon coated with Al_2_O_3_–chitosan	Single	Pb(II)	25	6	0.2	39.0	[198]

##### 5.4.4.4. Physical Modification

Physically modifying fruit peels exhibited variable biosorption behavior depending on the fruit peel type and metal ion, as shown in Table 13. Among the summarized biosorbents, watermelon rind showed notable Pb^2+^ sorption capacities up to 230.5 mg/g, attributed to their naturally high hydroxyl and carboxyl functional group content [36, 165]. Physically modified orange peels also demonstrated good adsorption performance, with adsorption capacities of 67.15 mg/g for Pb(II) and 54.94 mg/g for Cu(II). However, lower adsorption capacities were observed for Mn(II) (21.43 mg/g) and Co(II) (20.37 mg/g), indicating that extraction efficiency depends on the properties of the target metal ion. Similarly, dragon fruit peels showed varying adsorption capacities for different metals, with the highest adsorption recorded for Pb(II) (82.64 mg/g), followed by Cd(II) (17.95 mg/g), Co(II) (6.01 mg/g), and Ni(II) (5.32 mg/g). These differences show variations in the interaction between the naturally occurring functional groups on the fruit peel surface and different metal ions. Ultrasound treatment also enhanced the adsorption performance of citrus peels by improving surface accessibility of binding sites. Ultrasound‐treated lemon peels exhibited higher adsorption capacities for Ni(II) (14.31 mg/g) and Zn(II) (38.46 mg/g) than ultrasound‐treated orange peels, which adsorbed 5.45 mg/g and 14.64 mg/g, respectively.

**Table 13:** Comparison of physically modified fruit peel biosorbents.

Biosorbent	Type	Heavy metal ions	Optimum conditions			Adsorption capacity *Q* *m* *a* *x* (mg/g)	References
			Temp (°C)	pH	Dose (g)		
Orange peels	Single	Cu(II)	25	5.5	1	54.94	[206]
		Pb(II)		6		67.15	
		Mn(II)	25	5.42	0.2	21.432	[207]
		Co(II)		6.05		20.366	

Dragon fruit peels	Single	Co(II)	25	5.5	0.1	6.013	[165]
		Cd(II)				17.95	
						82.64	
		Pb(II)				5.322	
		Ni(II)					

Watermelon rind	Single	Pb(II)	25	5	1	230.5	[36]

Ultrasound‐treated lemon peels	Single	Ni(II)	25	4	1	14.31	[208]
		Zn(II)				38.46	
		Co(II)					
Ultrasound‐treated orange peels	Single	Ni(II)	25	4	1	5.45	
		Zn(II)				14.64	

The findings from the comparative evaluation of modified fruit peel biosorbents indicate that each modification strategy provides certain advantages depending on the target heavy metal ion and the desired adsorption performance. Chemical functionalization and metal‐oxide modification generally achieved the highest adsorption capacities. However, high adsorption capacity alone does not determine the suitability of a biosorbent for practical applications, as factors such as modification cost, environmental impact, preparation complexity, and scalability must also be considered. Therefore, future development of modified fruit peel biosorbents should focus on achieving a balance between adsorption efficiency and practical feasibility. Figure 3, demonstrates these factors for effective application of these modified biosorbents in industrial set‐ups.

**Figure 3:**
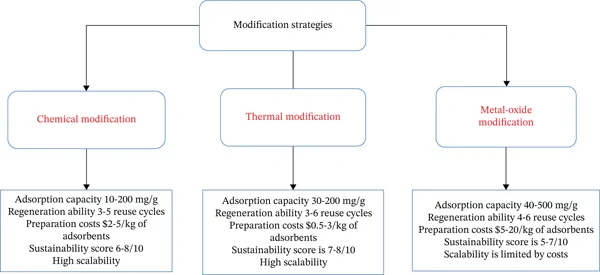
Quantitative comparison of the modification strategies of fruit peel biosorbents.

The adsorption capacities, regeneration ability, environmental friendliness, preparation costs, and scalability of fruit peel biosorbents are significantly influenced by the choice of modification. Figure 3, summarizes the quantitative comparisons of the reported modification strategies. From the summarized illustration, metal‐oxide modification provides the highest biosorption capacities (40–500 mg/g) resulting from additional active sites and improved surface properties. However, it has limited regeneration (4–6 reuse cycles), requires a higher preparation cost ($5–20/kg adsorbent), and has lower scalability because of material cost and possible metal leaching issues. Chemical modification, on the other hand, enhances sorption, reaching capacities of 10–200 mg/g, with a moderate regeneration ability (3–5 cycles), and lower preparation costs ($2–5/kg adsorbent). However, chemical treatments are not environmentally friendly because of the subsequent waste generation and the need for toxic reagents. Thermal modification strategies result in a higher biosorption efficiencies compared with chemical treatments with moderate regeneration abilities. Moreover, these thermally activated fruit peel biosorbents have proved environmentally friendly with a higher sustainability score of 7–8/10.

### 5.5. Mechanism of Biosorption

The mechanism of biosorption is explained by processes such as physisorption, involving electrostatic attraction, and chemisorption, which involves processes such as ion exchange, surface complexation, and precipitation [33]. In most cases, more than one mechanism can be involved in the biosorption process because of its complex nature [102]. The exchange of heavy metal ions with the ions that are present on the surface of the biosorbent is entailed in ion exchange [143, 209], whereas surface complexation involves the formation of complexes between the functional groups present on the biosorbent surface and the heavy metal ions [33]. Precipitation, on the other hand, involves the formation of insoluble complexes when the heavy metal ions react with the functional groups present on the surface of the biosorbent [209]. Physicochemical analysis and spectroscopic techniques allow for the decipherment of underlying mechanisms during the biosorption process [36]. Figure 4 describes the major biosorption mechanisms, facilitating the interactions between the fruit peel biosorbents and the heavy metal ions.

**Figure 4:**
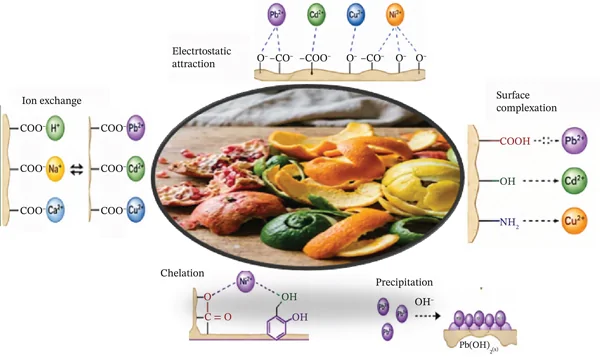
Schematic illustration of the biosorption mechanisms of fruit peel wastes [187].

Afolabi and Musonge [37] reported electrostatic attraction biosorption for the removal of Cu(II) and Pb(II) ions using banana peel powder as the biosorbent. This was revealed by the shift of the peaks in the FTIR spectra after the biosorption process. Similar findings were also reported by Praipipat and colleagues [31] for the biosorption of lead ions using both the orange peel powder and orange peel powder doped with iron (III) oxide‐hydroxide, showing that the modification process does not affect the sorption mechanism. The sorption process occurred from the donation of a proton from the functional groups present on the surface for an electrostatic interaction with the heavy metal ions. Phuengphai et al., [104] explained that the biosorption of Cu(II) ions, as shown by the pH effect and FTIR‐based characterization techniques, was supported by electrostatic attraction between the carboxylate group present on chemically modified rambutan, dragon fruit, and passion fruit peel with the metal ions, whereas Wang et al., [36] reported that electrostatic attraction only contributed a small part to the biosorption of Pb(II) ions by watermelon rind. The main mechanism involved in the biosorption process was an ion exchange between Mg^2+^ or Ca^2+^ ions released from the watermelon rind and the lead ions. Micro‐precipitation of lead(II) hydroxide was also observed on the surface of the watermelon rind, indicating a possible mechanism for the biosorption process.

According to Wattanakornsiri et al., [174], the mechanism of the biosorption of lead and cadmium ions from wastewater by chemically modified rambutan, dragon fruit, and passion fruit peel waste is based on ion exchange between the carboxyl, phenolic, hydroxyl, and other functional groups of lignin, tannin, or cellulose, which are the major components of the materials and the metal cations. The FTIR analysis confirmed the ion exchange mechanism of interaction between the heavy metal ions and the biosorbents. A similar mechanism of biosorption was also reported by Jahin et al., [182] in the removal of copper and zinc ions using chemically activated waste banana peels.

Apart from the FTIR analysis, other characterization techniques such as x‐ray photoelectron spectroscopy (XPS), extended x‐ray absorption fine structure (EXAFS), scanning electron microscopy (SEM), and BET are employed in the analysis of the biosorption mechanism. Chemical complexation is revealed by XPS and EXAFS, whereas SEM and BET describe the physical biosorption of the heavy metal ions [210]. For instance, the change of the spectra in XPS and XRD indicated that the mechanism of the biosorption of Pb(II) and Cu(II) metal ions by thermally modified magnetic pomelo peels is chemical complexation and electrostatic attraction between the biochar and the heavy metal ions [211]. Additionally, the XPS spectra for chemically modified orange peels proved Cd‐sulfhydryl complexation as the dominant mechanism in the biosorptive removal of Cd(II)ions from synthetic solutions [212]. Moreover, solid state NMR has been employed to identify the functional groups and the molecular structures of the compounds present in fruit peel biosorbents [213].

### 5.6. Factors Affecting the Biosorption Process

The biosorption process is influenced by several parameters, including pH of the solution, contact time, biosorbent dose, temperature, and the metal ion concentration.

#### 5.6.1. Biosorbent Dose

Biosorbent dose is a critical factor in the biosorption process, providing active sites for the binding of heavy metal ions. According to studies, there is a direct relationship between the biosorbent dose and the removal efficiency; that is, increasing the biosorbent dose increases the removal efficiency of heavy metal ions and vice versa [162]. This increase in the removal efficiency is attributed to an increase in the oxygen‐containing functional groups as the biosorbent dose is increased [162]. For instance, an increase in the orange peel powder doped with iron (III) oxide‐hydroxide from 1 to 6 grams increased the lead removal efficiencies from 90% to 97.86% with an optimum dose of 3 grams [31]. Similarly, increasing the banana peel biosorbent dose from 0.1 to 0.5 grams increased the removal efficiency of copper ions from 33% to 60% [182]. Conversely, an increase in the adsorbent dose can also have minimal or no influence on the removal efficiencies. Jahin et al. reported that an increased dose of the banana peel biosorbent between 0.5 and 0.6 grams resulted in minimal improvement in removal efficiency because of the agglomeration of the banana peel particles at higher doses, thereby decreasing the available sorption sites [182].

#### 5.6.2. Influence of pH

The pH of the solution affects the surface charge on the biosorbent as well as the metal ions speciation, which in turn impacts the sorption capacity [36]. For example, Cr(VI) ions predominate in solution as HCrO−_4_ ions at low pH values of around 2, which are smaller in size, allowing for easy adsorption [163]. pH also influences the competitive adsorption of heavy metal ions on the biosorbent surface due to increased hydrogen ion concentration [181]. Increasing the pH results in less hydrogen ion competition for the binding sites and the deprotonation of weakly acidic functional groups such as carboxyl groups, providing sites for higher removal efficiencies [36]. In a study by Praipipat et al., [31] it was shown that orange peel powder and the orange peel powder doped with iron (III) oxide‐hydroxide were effective for lead removal when pH values increased from 1 to 5, with the optimal pH at 5, leading to higher lead removal efficiencies. This was because of a higher presence of hydrogen ions on the biosorbent′s surface at the lower pH values, which limited lead adsorption. For the biosorption of Cu(II) and Zn(II) ions using banana peel biosorbent, removal efficiencies rose from 30% to 40% for copper ions and from 42% to 58% for zinc ions as the pH increased from 3 to 6, with the optimal pH at 6 [182].

Moreover, the pHpzc of fruit peel biosorbents strongly influences the pH range for the biosorption of heavy metal ions. The pHpzc is the pH at which the surface of the biosorbent is neutral, and the pH below the pHpzc results in a positively charged surface that repels metal ions, whereas a pH above the pHpzc results in a negatively charged surface favorable for the adsorption of heavy metal ions [36]. For example, the pHpzc value for pomelo peels doped with zinc oxide was found as 7.19, showing that favorable removal efficiencies of the metal ions could only occur at pH above 7.19 [202]. Similarly, the pHpzc value for orange peels was reported as 2.63, meaning that a pH value above that increases the removal rate, such as the 97.68% removal rate for Cr(VI) ions that was obtained at a pH of 10 [163]. Still, at these higher pH values, precipitation of metal ions occurs as a mechanism of their removal.

#### 5.6.3. Role of Contact Time

The effect of contact time is essential for understanding the reaction kinetics and identifying the equilibrium point in an adsorption process [169]. Understanding the reaction kinetics is critical as it determines the reaction rate, the rate determining step, and the processes involved in the biosorptive uptake of heavy metal ions [214]. This kinetic process involves the transfer of metal ions from the bulk solution phase to the biosorbent′s surface which is often limited by the resistance from the liquid film surrounding the biosorbent′s particles, followed by the movement of ions through the pores and finally the binding step [214]. Initially, the removal efficiency of heavy metal ions is high because of the availability of vacant active sites, but as contact time increases, these sites become saturated, reaching an equilibrium on the biosorbent surface [169]. For example, varying contact time between 30 and 210 min resulted in a maximum removal efficiency of 72% for Cu(II) ions at 150 min and 80% for Zn(II) ions at 180 min, using banana peel biosorbent [182]. Further increases in contact time did not improve the removal efficiency of Cu(II) and Zn(II) [182] ions because of a lack of available binding sites. Similarly, the cadmium removal efficiency of banana powder beads increased when contact time was extended from 1 to 8 h, with the highest removal efficiency of 89.54% at 8 h [201].

#### 5.6.4. Metal Ion Concentration

The initial metal ion concentration is another critical factor that significantly influences the biosorption process of heavy metal ions. This is because it determines the mass transfer driving force of the heavy metal ions between the solution and the biosorbent′s surface. Most recent studies report that increasing the initial metal ion concentration increases the adsorption capacity while reducing the overall removal efficiencies of heavy metal ions based on the biosorbent′s properties and the process conditions. An increase in the metal ion concentration generally develops a concentration gradient that increases the driving force of the metal ions towards the biosorbent′s surface, thereby increasing the adsorption capacity [105].

Fruit peel biosorbents exhibit a higher biosorptive uptake of heavy metal ions at lower initial metal ion concentrations, as numerous active sites are available on the adsorbent surface. For instance, rambutan peels demonstrated higher removal efficiencies (98.80%) of Cu(II) ions at lower initial concentrations of 100 mg/L which reduced to 86% with an increase in the concentration to 500 mg/L [104]. Additionally, the extraction efficiencies of dragon and passion fruit peels sharply decreased from 71% and 81%, respectively, at 100 mg/L to 46% and 57%, respectively, at 500 mg/L [104]. This was attributed to the abundance of functional groups at the lower initial metal ion concentrations that readily bonded with the metal cations [209]. Moreover, increasing the initial cadmium ion concentration from 25 to 200 mg/L resulted in decreased removal efficiencies of banana powder beads, and the beads doped with ZnO and MgO [201], whereas the removal efficiency of Pb(II) ions also decreased from 95.31 to 68.35% when the initial metal ion concentration was increased from 50 to 200 mg/L [215]. In general, such biosorption behavior is attributed to an unchanging number of the available active sites at a constant adsorbent dose relative to an increasing amount of sorbates [174].

#### 5.6.5. Effect of Coexisting Ions

Apart from heavy metal ions, wastewater contains a variety of coexisting anions and other pollutants that can hinder the biosorption process. Thus, assessing their effect in the biosorptive removal of heavy metal ions is essential in the industrial scale up of fruit peel biosorbents. The effect of Ca^2+^, Na^+^, Cl^−^, NO_2_^−^/NO_3_^−^, HCO_3_^−^, PO_4_^3−^, and SO_4_^2−^ ions on the biosorptive removal of As(V) was assessed using chemically modified pomelo peels [147]. The findings demonstrated that these co‐existing ions reduced the biosorptive uptake of As(V) based on the charge and binding affinities in the order of $\text{P}{{\text{O}}_{4}}^{3-}>\text{S}{{\text{O}}_{4}}^{2-}>\text{C}{{\text{O}}_{3}}^{2-}>\text{N}{{\text{O}}_{2}}^{-}/\text{N}{{\text{O}}_{3}}^{--}\approx \text{C}{\text{l}}^{-}>\text{N}{\text{a}}^{+}\approx \text{C}{\text{a}}^{2+}$ [147]. A similar interference trend ($\text{S}{{\text{O}}_{4}}^{2-}>\text{N}{\text{O}}_{3}>\text{C}{\text{l}}^{-}$) was also observed in the biosorptive removal of As (III) ions using saponified mango peels loaded with Zr(IV) ions [216]. This interference trend was attributed to the competition of active sites and the formation of highly stable complexes, reducing the overall extraction efficiencies [216]. However, any concentration level of SO_4_^2−^ and Cl^−^ had minimal interference in the biosorptive uptake of As(III) ions using Fe(III) loaded saponified pomegranate peels, as they were less capable of binding with the cations in solution [217]. The presence of PO_4_^3−^ ions, on the other hand, significantly binds with the arsenic cations, forming complexes and reducing the overall sorption uptake [217].

Table 14 summarizes the influence of various biosorption parameters on the removal efficiencies and adsorption capacities of heavy metal ions using fruit peel biosorbents. It demonstrates the effect of the process parameters on the biosorption process, the general trend that is reported in the literature, and some of the example findings reported in recent studies. All of the reported factors directly influence the biosorption performance, and the optimization results provide room for scale‐up. However, different operational parameters are reported for various fruit peel biosorbents; thus, future research should focus on comparatively analyzing the influence of these process parameters for various fruit peel biosorbents to understand their superiority in biosorption performance. Additionally, more studies should focus on the influence of these process parameters on the adsorption of other underexplored toxic metal ions such as uranium, thallium, antimony, and vanadium ions.

**Table 14:** Influence of the various parameters on the biosorption performance.

Parameter	Effect	General trend reported in literature	Literature findings	Ref
Solution pH	Influences the biosorbent′s surface charge and the metal ion speciation.	Increase in the extraction efficiency with an increase in pH	Dinh et al. reported that uptake of Pb(II) and Cd(II) ions increased with the solution pH from 2 (< 20%) to 5 (> 80%), reaching the maximum uptake at 5.5. Further increase in the pH (a pH of 6) resulted in hydroxide precipitation forming Pb(OH)_2_^+^ and Cd(OH)_2_^+^ species.	[167]
		Lower pH conditions (< 3) result in H^+^ ions competition for active sites while higher pH conditions (> 7) cause ion precipitation		
Initial metal ion concentration	Influences the mass transfer driving force of the heavy metal ions between the solution and the biosorbent′s surface	Increasing the initial metal ion concentration increases the adsorption capacity while reducing the removal efficiencies	Wattanakornsiri et al. reported that the adsorption capacities of Pb(II)and Cd(II) metal ions using rambutan, dragon, and passion fruit peels increased with an increase in the ion concentration (from 100–500 mg/L), while the percentages of metal removal from the aqueous solution for DFP, RP and PFP slightly decreased from 92.90%–56.04%, 87.10%–64.32%, 94.50%–61.60% for Pb(II), and 95.29%–51.62%, 99.57%–53.83%, 83.50%–56.20% for Cd(II).	[174]
Contact time	Influences the kinetics of the biosorption process	Increasing the contact time between the metal cations and the fruit peel biosorbents provides more interactions, resulting in higher removal efficiencies	Increasing the contact time (1–72 h) between the banana and orange beads biosorbents with a cocktail solution containing Cd(II), Cu(II), Hg(II), and Ni(II), increased the removal efficiencies from < 20% to > 80% until a stable optimum was achieved	[218]
Adsorbent dose	Provides active sites for efficient extraction of heavy metal ions	Increasing the amount of the biosorbent provides additional binding sites for heavy metal ions chelation, resulting in higher extraction efficiencies	Jahin et al. report an increase in the removal efficiency of Cu(II) ions from 33 to 60% with an increase in the banana peels dose from 0.1 to 0.5 grams.	[182]
			However, further increase in the dose (to 0.6 grams) resulted in the agglomeration of the banana peel particles, reducing the overall efficiency.	
Coexisting ions	Competition for active sites	Coexisting ions tend to form stable complexes with the metal cations, reducing the removal efficiencies	PO₄^3−^ and SO₄^2−^ significantly reduced As(V) adsorption using Fe(III)‐modified pomelo peels, whereas Cl^−^ and NO₃^−^ had negligible effects. The interference followed the order PO₄^3−^ > SO₄^2−^ > HCO₃^−^ > NO₃^−^ ≈ Cl^−^.	[147]
		These ions also compete for the biosorbent′s active sites, reducing the overall extraction rates		
Heavy metal ion type	Depends on the ionic radius, electronegativity, and complexation behavior	For most of the studied heavy metal ions, Pb(II) ions have demonstrated the highest adsorption affinities for various fruit peel biosorbents	Pomegranate peels demonstrated a higher binding affinity for Pb(II) ions as revealed by the higher extraction capacities of 5.985 mg/g (single metal system) and 1.378 (multimetal system) compared to Cd(II), Co(II), and Ni(II) metal ions that reported adsorption capacities of 4.482, 1.728, and 1.455 mg/g, respectively, in single metal systems and 1.235, 0.441, and 0.378 mg/g, respectively, in multimetal systems	[165, 167, 174]

### 5.7. Kinetics and Isotherm Studies

Adsorption isotherms measure the biosorption process at a specific temperature by linking the amount of heavy metal ions retained by the biosorbent to their concentration in solution at equilibrium [83]. They are crucial for understanding the mechanism of adsorption and for finding the maximum adsorption capacity of an adsorbent [49]. Freundlich and Langmuir isotherm models are the two common empirical models used to describe biosorption at a given temperature [83]. For example, the adsorption pattern of orange peel powder and orange peel powder doped with iron (III) oxide‐hydroxide followed the Freundlich isotherm model, as indicated by its highest *R* ^2^ values compared to the Langmuir, Temkin, and Dubinin‐Radushkevich models relating to physicochemical adsorption [31].

Similar findings were also reported by Praipipat et al. [201], for the biosorption of cadmium ions using metal oxide‐doped banana powder beads, which suggested a multilayer sorption process. In another related study, the biosorption of Cu(II) and Zn(II) metal ions using banana peel biosorbent fitted the Langmuir isotherm more than the Freundlich model, indicating monolayer adsorption on the biosorbent′s surface [182]. The Langmuir isotherm model also described the biosorption of Cu(II) ions utilizing rambutan, dragon fruit, and passion fruit peel biosorbent [104]. Similar results were also reported by Wattanakornsiri and coworkers [174], for the biosorption of Pb(II) and Cd(II) ions using similar fruit peel biosorbents. Kinetic models, on the other hand, mainly describe the removal rate of heavy metal ions during the biosorption process [83]. For the kinetic studies, the pseudo‐second‐order model has been reported as the best fit for all the fruit peel biosorbents, modified or unmodified, for the biosorption of heavy metal ions [31, 104, 174, 182, 201].

The kinetic and isotherm models for various fruit peel biosorbents have been summarized in Table 15. Most studies report pseudo‐second order as the kinetic model, describing chemical interactions between the heavy metal ions and the functional groups on the surface of the fruit peel biosorbents [201]. Very few studies have reported pseudo‐first order kinetic model that suggests the interaction between the fruit peel biosorbents and the heavy ions occurring through physical forces. Furthermore, Langmuir isotherm has been reported as the common isotherm model, describing monolayer adsorption of the heavy metal ions on the biosorbent′s surface [182, 220]. The heterogeneity of surfaces has also been described in other studies, which provided Freundlich isotherm as the best fit in the biosorption experiments.

**Table 15:** Kinetic and isotherm models of various fruit peel biosorbents.

Fruit peel	Heavy metal ions	Type	Optimized Conditions					Maximum capacity *Q* *m* *a* *x* (mg/g)	Kinetic model	Isotherm model	Ref
			pH	Time (min)	Dose (g)	Ion conc (mg/L)	Temp (°C)				
Chemically modified pomegranate peel	Cr(VI)	Single	2	180	0.025	50	25	82.99	Pseudo‐second order	Langmuir	[158]
*Pachira aquatica* Aubl. fruit peel biochar	Ni(II)	Multielement	5	180	0.5	70	30	5.65	Pseudo‐second order	Langmuir	[194]
	Cd(II)							30.44			
Fe(III)‐modified pomelo peels	As(V)	Single	2	120	0.04	4.65	25	64.43	Pseudo‐second‐order	Langmuir	[147]
Tommy Atkins mango peels	Pb(II)	Single	5.5	60	0.05	100	25	36.7	Pseudo‐first order	Langmuir	[219]
H_2_SO_4_‐modified banana peels	Cr(VI)	Single	2	—	1	100	30	161	Pseudo‐second order	Langmuir	[175]
Ultrasound treated lemon peels	Ni(II)	Single	4	30	0.5	5	25	5.45	Pseudo‐second order	Langmuir for Ni(II) and Freundlich for Zn(II)	[208]
	Zn(II)			60		10		14.64			
Watermelon rind	Pb(II)	Single	5	1440	1	500	25	230.5	Pseudo‐first order	Freundlich	[36]

### 5.8. Thermodynamic Studies

To comprehend the practicality of these fruit peel biosorbents in wastewater treatment and their reusability potential for industrial applications, it is necessary to understand the thermodynamics of the sorption process [221]. The feasibility of the biosorption process is explained by thermodynamic parameters, including the change in enthalpy, entropy, and Gibbs free energy [222]. The Gibbs free energy change (*Δ*G^°^) describes the favorability and spontaneity of the biosorption process, with values < 0 reflecting a spontaneous process while values > 0 demonstrating a nonspontaneous process [223]. The biosorptive removal of heavy metal ions using fruit peel biosorbents has been reported as thermodynamically favorable, revealed from the negative Gibbs free energy change obtained from various studies [175, 194, 219, 224, 225]. For instance, the biosorptive removal of Pb(II) ions using banana peels reported negative values of *Δ*G^°^ (−3.88, −4.79, −5.95, −6.44, −7.41, and −8.50 kJ/mol) at all the selected temperatures (283, 288, 293, 298, 303, and 308 K) [226]. Similar findings were also reported when chemically modified pomelo peels were utilized in the biosorptive removal of As(V) from synthetic solutions [147]. The study reported *Δ*G^°^ values of −18.40, −19.98, and −21.01 kJ/mol at 298, 303, and 308 K, respectively, demonstrating the favorability of the biosorption process.

Apart from changes in enthalpy (*Δ*H^°^), the influence of temperature on the spontaneity of the biosorption process indicates whether the process is exothermic or endothermic [227]. An increase in spontaneity with temperature indicates an endothermic biosorption process, whereas a decrease indicates an exothermic biosorption process [227]. For the biosorptive removal of Pb(II) ions using banana peels, the *Δ*G^°^ values decreased with temperature (from −3.88 kJ/mol at 283 K to −8.50 kJ/mol at 308 K), revealing that higher temperatures increased the spontaneity of the biosorption process [226]. This described an endothermic biosorption process, which was consistent with the positive *Δ*H^°^ values obtained (55.85 kJ/mol). Similarly, the use of chemically modified pomelo peels demonstrated an increase in spontaneity with temperature as the *Δ*G^°^ decreased from −18.40 kJ/mol at 298 K to −21.01 kJ/mol at 308 K [147]. This was also consistent with the reported *Δ* *H*° value (58.24 kJ/mol).

However, for the biosorptive removal of Pb(II) ions using Tommy Atkins mango peels, the *Δ*G^°^ increased with temperature (from −9.08 kJ/mol at 278.15 K to −2.64 kJ/mol at 318.15 K) describing an exothermic biosorption process [219]. This demonstrated nonspontaneity at higher temperatures and was consistent with the reported *Δ*H^°^ of −53.97 kJ/mol. Similar findings were also reported for the biosorptive removal of Zn(II) ions using raw and acid‐modified orange peels [224]. It is also reported that the modification of fruit peel biosorbents influences the spontaneity of the biosorption process. Parashar et al. demonstrated that modifying orange peels using H_2_SO_4_ provided a higher *Δ*G^°^ (−6.71 kJ/mol) for the sorption process compared to the *Δ*G^°^ reported for the raw orange peels (−4.73 kJ/mol) [224]. This indicated that spontaneity of the biosorption process increased with the acid treatment. Similar results were also observed when pomegranate peels were pyrolyzed and chemically activated using ZnCl_2_ to provide activated carbon for the biosorptive removal of lead ions [225]. The *Δ*G^°^ increased from −16.306 kJ/mol for the raw pomegranate peels to −16.635 kJ/mol for the activated carbon at 298 K, increasing spontaneity. However, raw pomegranate peels demonstrated a higher *Δ*G^°^ of −16.306 kJ/mol at 298 k compared with the biochar obtained from the peels, which demonstrated a *Δ*G^°^ of −15.919 kJ/mol at 298 K.

Entropy describes the randomness of a biosorption process, with a decrease in randomness often demonstrated by negative changes in entropies (*Δ*S^°^), whereas positive changes describe an increase in randomness [224]. The biosorptive removal of heavy metal ions using fruit peel biosorbents is often accompanied by positive changes in entropy. This randomness observed at the adsorbate‐adsorbent interface is influenced by the release of some ions in solution during the biosorption process [147]. For example, the removal of Cr(VI) ions from aqueous solutions using acid‐modified banana peels demonstrated a positive *Δ*S^°^ of 77.6 J/mol K, indicating an increase in randomness during the sorption process [175]. Moreover, the biosorptive removal of Ni(II) and Cd(II) ions also demonstrated an increase in randomness with the *Δ*S^°^ values reported as 14 and 34 J/mol K for the removal of Ni(II) and Cd(II), respectively [194].

### 5.9. Application in Real Wastewater Samples

The complex nature of wastewater significantly influences the efficient biosorption of heavy metal ions using fruit peel biosorbents. This is attributed to the subsequent competition by other coexisting ions and blockage of pores by other pollutants that reduces the biosorbents extraction capacities [228]. The effective removal of Cr(VI) ions from textile effluent samples using nutmeg fruit peel wastes demonstrated that there was a slight decline in the extraction capacities attributed to the simultaneous coadsorption of the other ions in solution [228].

Recent studies have demonstrated that banana peel biosorbents, particularly after chemical or thermal modification, exhibit promising potential for the removal of toxic heavy metal ions from real wastewater. However, their adsorption performance is often lower than that observed in synthetic aqueous solutions. Jahin et al. investigated the application of chemically activated banana peels for the removal of Cu(II) and Zn(II) ions from wastewater [182]. The study reported a decline in the adsorption of Cu(II) ions, with the extraction efficiency decreasing from 72.0% in synthetic aqueous solutions to only 8.33% in wastewater. This reduction was attributed to competitive adsorption and interference from the other ions in the solution that occupied the available adsorption sites. Furthermore, the modification strategy also influences the extraction efficiency of the heavy metal ions in wastewater. Thermally activated banana peels demonstrated an 86.5% removal rate for Cu(II) ions from municipal wastewater, which was higher than that observed for the chemically treated peels [229].

Despite the lower extraction efficiencies for Cu(II) ions, the chemically activated banana peels exhibited varying removal efficiencies for other metal ions, achieving 95.83% for Pb(II), 79.80% for Sr(II), 62% for Cd(II), 54.73% for Mn(II), 28.50% for Fe(II), 24.49% for Se(II), 14.29% for Mo(II), and only 6.52% for Cr(II) [182]. Moreover, the chemically activated biosorbent removed 93.5% of Pb(II), 89% of Ni(II), 87.9% of Fe(II) and 79.5% of Cd(II) that were present in hospital wastewater [230]. These findings demonstrated that chemically modified banana peels possessed a greater affinity for Pb(II), which showed that the adsorption selectivity was dependent on the physicochemical properties of the heavy metal ions [182, 230]. The findings also described the broader application of chemically activated peels for the efficient elimination of toxic heavy metal ions from industrial wastewater. Thermally activated banana peels have also demonstrated high removal efficiencies of Pb(II) (92.4%) and Cd(II) (89.9%) from industrial wastewater comparable to commercial activated carbon that is commonly utilized in industries [7].

Although fruit peel biosorbents have demonstrated promising results in the elimination of heavy metal ions from wastewater, critical gaps still remain. Recent research has focused on the biosorptive use of banana peels for the removal of heavy metal ions from wastewater with little focus on the application of the other fruit peel waste. Most investigations involving other fruit peels as biosorbents have been conducted using synthetic aqueous solutions that do not accurately reflect the complexity of real water samples. In addition, most recent studies report lab‐scale batch adsorption experiments; thus future studies should employ continuous flow experiments and pilot‐scale wastewater treatment systems.

### 5.10. Regeneration of Biosorbents

The possible reuse of fruit peel waste biosorbents is important in the estimation of costs and understanding their economic viability for industrial applications. Regenerating these biosorbents not only reduces the need for new materials but also addresses environmental concerns related to their disposal [231]. Regeneration techniques include solvent washing, thermal treatment, chemical processes, and electrochemical regeneration [231]. The most common regeneration technique utilized in desorption studies involves the use of inorganic acids such as HCl, HNO_3_, and H_2_SO_4,_ which recover the bound metal cations through a proton exchange process [232]. However, these acids can damage the surface of the biosorbent to some extent, resulting in a reduction in the biosorption capacity [232]. Additionally, the reduction in the biosorption capacity can be attributed to irreversible chemisorption, physical pore blockage, or the structural degradation of the biosorbents from harsh acid washing [232]. Interestingly, orange peel powder and orange peel powder doped with iron (III) oxide‐hydroxide could be reused up to 5 cycles, utilizing 0.5 M HNO_3_ solution with high adsorption in the range of 55.14%–70.17% and 86.57%–96.64%, respectively, and high desorption for lead ions in the ranges of 50.06%–68.09% and 80.20%–94.23%, respectively [31]. Similar results were also reported by Villen‐Guzman et al. [233]. After five consecutive cycles of adsorption and desorption using 0.1 M of HNO_3_ and H_2_SO_4,_ a 90% recovery of Ni(II) ions was obtained.

Other studies have reported a decrease in the biosorption capacity of fruit peel wastes during desorption caused by the permanent binding of heavy metal ions during the previous cycles. Abatal and coworkers, [165] for example, reported that the sorption capacity from the first to the second cycle decreased from 6.56–0.71 mg/g, 6.107–4.102 mg/g, 1.630–1.462 mg/g, and 1.131–0.813 mg/g for the removal of Pb(II), Cd(II), Co(II), and Ni(II) ions, respectively. The removal efficiency for Cr (VI) ions also decreased from 94% to 58% from the first to the seventh run, when chemically activated biochar derived from waste banana peels was utilized as a biosorbent [197]. Similar results were also reported by Bai et al., [187] where after 5 cycles, the removal rate of lead ions by chemically modified hydrothermal carbon from waste banana peels decreased from 86.84% to 76.88%.

Overall, studies on the regeneration potential of fruit peel biosorbents demonstrate that their reusability is influenced by the desorption agent used and the nature of the adsorbed metal ions [234]. Studies have focused on the use of weak inorganic acids as desorption agents, demonstrating subsequent extraction capacities of between 60%–80% after 3–5 regeneration cycles [180, 182, 191, 218, 235, 236]. Additionally, the structural stability, characterized using techniques such as FTIR and SEM, strongly influences the regeneration performance. Modified fruit peel biosorbents have been reported to exhibit better structural stabilities than unmodified fruit peels that enhance their regeneration potential [236].

### 5.11. Life Cycle Assessment (LCA) of Fruit Peel Biosorbents

Fruit peel biosorbents should be assessed from a sustainability and mechanistic standpoint in addition to evaluating their heavy metal ion extraction efficiencies [171]. By taking into account the stages of peel sourcing, processing, modification, biosorption process, regeneration, and disposal, LCA has been found to be a crucial tool for assessing these biosorbents environmental performance [171]. According to recent research, fruit peel adsorbents can lessen environmental burdens by promoting circular economy strategies and valorizing agricultural wastes [115]. However, their overall sustainability benefits may be diminished by energy‐intensive processing, chemical activation, and poor management and disposal of spent adsorbents. Simultaneously, because adsorption is pH‐dependent, surface complexation modeling (SCM) is becoming widely acknowledged as a useful tool for comprehending the mechanisms controlling metal uptake by fruit peel materials.

## 6. Perspective and Future Directions

Most research has concentrated on lab‐scale studies rather than real‐world wastewater applications. Although aqueous solutions provide controlled conditions for testing, they do not fully reflect the complex composition of wastewater, which contains various ions and contaminants. Future research should investigate the biosorption capabilities of modified fruit peel biosorbents for removing heavy metal ions from actual water samples to assess their practical effectiveness. The long‐term stability of these biosorbents should also be evaluated through continuous flow experiments. Furthermore, advanced analytical techniques should be employed in future studies to understand the changes occurring during the modification process of fruit peel waste biosorbents. Proximate and ultimate analyses should also be included to determine the composition of cellulose, hemicellulose, pectin, and lignin in modified fruit peel wastes. Additionally, fruit peel wastes could serve as synthesis agents for various nanoparticles, especially for heavy metal ion sorption, because of their rich bioactive composition that may act as active sites and should be explored further. Emerging research directions, such as the application of machine learning for adsorbent design, artificial intelligence‐assisted adsorption prediction, computational chemistry, molecular simulations, techno‐economic assessment, and carbon footprint or life‐cycle analysis, should also be explored. They will highlight future opportunities and facilitate the practical translation of fruit peel‐based biosorbents from laboratory research to industrial applications.

## 7. Conclusion

The presence of heavy metal ions in water sources raises apprehension because of their potential toxicity to both humans and the environment. The removal strategies currently employed often suffer several limitations, including high costs, limited selectivity, reduced efficiency in highly concentrated solutions, and the creation of secondary pollutants. Using fruit peel wastes as sustainable alternatives is cost‐effective, and modification methods significantly improve their adsorption capacity. This review has tackled fruit peel wastes as biosorbents for heavy metal ion removal, the modification strategies currently employed, the mechanism of the biosorption process, their regeneration ability, and their potential as modification agents for the biosynthesis of nanoparticles, and the following conclusions can be drawn. The composition of fruit peel wastes containing various functional groups makes them efficient, low‐cost alternatives for the removal of various heavy metal ions from water and wastewater. Modifying fruit peel wastes through physical, chemical, thermal, and metal‐oxide modification strategies enhances the biosorption capacities but poses additional challenges. Among these modification strategies, metal‐oxide modification has proven very effective. However, it results in additional costs, questioning their economic viability, and the metal oxides can contaminate water sources, causing water pollution. Additionally, the main mechanisms involved in the biosorption process are associated with electrostatic attractions, ion exchange, and surface complexation. These biosorbents can easily be regenerated and used in subsequent adsorption cycles, but an LCA needs to be evaluated. Furthermore, most of the modified fruit peel biosorbents are applied in synthetic water samples, which cannot fully capture the complex nature of wastewater.

**Nomenclature**
BET: Brunauer–Emmett–Teller
EPA: Environmental Protection Agency
FTIR: fourier‐transform infrared spectroscopy
NMR: nuclear magnetic resonance
SCM: surface complexation modeling
SDGs: Sustainable Development Goals
SEM: scanning electron microscopy
WHO: World Health Organization
XFAS: x‐ray absorption fine structure
XRD: x‐ray diffraction

## Author Contributions

George S. Nyamato and Abiegael N. Kiiru conceptualized the work. Abiegael N. Kiiru wrote the first draft of the manuscript. George S. Nyamato, Joanne Ogunah, and Stephen O. Ojwach reviewed the draft manuscript and commented on previous versions of the manuscript.

## Funding

No funding was received for this manuscript.

## Disclosure

All authors read and approved the final manuscript.

## Ethics Statement

The authors have nothing to report.

## Consent

The authors have nothing to report.

## Conflicts of Interest

The authors declare no conflicts of interest.

## Data Availability

Data sharing is not applicable to this article as no datasets were generated or analysed during the current study.
